# Swd2/Cps35 determines H3K4 tri-methylation via interactions with Set1 and Rad6

**DOI:** 10.1186/s12915-024-01903-3

**Published:** 2024-05-03

**Authors:** Junsoo Oh, Shinae Park, Jueun Kim, Soojin Yeom, Ji Min Lee, Eun-Jin Lee, Yong-Joon Cho, Jung-Shin Lee

**Affiliations:** 1 Department of Molecular Bioscience, College of Biomedical Science, Kangwon National University, Chuncheon, 24341 Republic of Korea; 2 Institue of Life Sciences, Kangwon National University, Chuncheon, 24341 Republic of Korea; 3 Kangwon Institute of Inclusive Technology, Kangwon National University, Chuncheon, 24341 Republic of Korea; 4 Graduate School of Medical Science & Engineering, Korea Advanced Institute of Science and Technology, 291 Daehak-Ro, Yuseong-Gu, Daejeon, 34141 Republic of Korea; 5 Department of Life Sciences, Korea University, Seoul, 02841 Republic of Korea; 6 Multidimensional Genomics Research Center, Kangwon National University, Chuncheon, 24341 Republic of Korea

**Keywords:** H3K4 trimethylation, H2B ubiquitination, Rad6, Set1, Swd2

## Abstract

**Background:**

Histone H3K4 tri-methylation (H3K4me3) catalyzed by Set1/COMPASS, is a prominent epigenetic mark found in promoter-proximal regions of actively transcribed genes. H3K4me3 relies on prior monoubiquitination at the histone H2B (H2Bub) by Rad6 and Bre1. Swd2/Cps35, a Set1/COMPASS component, has been proposed as a key player in facilitating H2Bub-dependent H3K4me3. However, a more comprehensive investigation regarding the relationship among Rad6, Swd2, and Set1 is required to further understand the mechanisms and functions of the H3K4 methylation.

**Results:**

We investigated the genome-wide occupancy patterns of Rad6, Swd2, and Set1 under various genetic conditions, aiming to clarify the roles of Set1 and Rad6 for occupancy of Swd2. Swd2 peaks appear on both the 5ʹ region and 3ʹ region of genes, which are overlapped with its tightly bound two complexes, Set1 and cleavage and polyadenylation factor (CPF), respectively. In the absence of Rad6/H2Bub, Set1 predominantly localized to the 5ʹ region of genes, while Swd2 lost all the chromatin binding. However, in the absence of Set1, Swd2 occupancy near the 5ʹ region was impaired and rather increased in the 3ʹ region.

**Conclusions:**

This study highlights that the catalytic activity of Rad6 is essential for all the ways of Swd2’s binding to the transcribed genes and Set1 redistributes the Swd2 to the 5ʹ region for accomplishments of H3K4me3 in the genome-wide level.

**Supplementary Information:**

The online version contains supplementary material available at 10.1186/s12915-024-01903-3.

## Background

Trimethylation of histone H3 lysine 4 (H3K4me3) is considered a hallmark of promoters of actively transcribed genes across diverse organisms from yeast to humans [1, 2]. Dysregulation of H3K4me3 and its modifiers has been linked to cancer pathogenesis and aberrant development in mammals, underscoring the importance of understanding its regulatory mechanisms [3–6]. The H3K4 methylation process, involving Set1/COMPASS methyltransferase, is highly conserved among eukaryotes, making budding yeast a valuable model for its study [1, 4]. Yeast Set1/COMPASS comprises Set1, an essential catalytic component, and seven accessory proteins, each contributing to protein stability or H3K4 methylating activity [7–10]. Swd2/Cps35 is the only essential component in Set1/COMPASS, because of its function within the cleavage and polyadenylation factor (CPF) transcription termination complex [9, 11, 12].

H3K4me3, as well as dimethylation, is dependent on monoubiquitination at the histone H2B (H2Bub) in yeast, fruit flies, and mammals [13–16]. A previous study has shown that Swd2/Cps35’s binding to the rest of Set1/COMPASS is reduced in the absence of H2Bub and Swd2 addition to defective Set1/COMPASS, which was isolated from a strain deficient in H2Bub, enhances its in vitro H3K4me3 activity, emphasizing the importance of H2Bub-dependent binding of Swd2 to the rest of the Set1/COMPASS [17]. This previous study also showed that Swd2, rather than the rest of the Set1/COMPASS, was recruited by H2Bub into transcribed genes and facilitated the assembly of H3K4me3-competent Set1/COMPASS [17]. However, contrasting findings emerged by other groups: Rad6/H2Bub appeared necessary for Set1 and Swd2 occupancy on transcribed genes, with Set1 essential for Swd2 occupancy as well [11, 17]. These conflicting findings regarding Rad6/H2Bub’s role in the occupancy of both Set1 and Swd2 on transcribed genes have raised questions, possibly due to variations in gene selection for chromatin immunoprecipitation (ChIP-qPCR) [11, 17].

In this study, we investigated the chromatin occupancy of Set1, Swd2, and Rad6 at the genome level. ChIP-seq data revealed that occupancy of the Swd2 appears on the transcribed genes with specific enrichments on both 5ʹ region and 3ʹ region of genes, which might be mediated through the tight interactions with Set1/COMPASS and CPF, respectively [7–9, 12]. Our results indicated that in the absence of the Rad6/H2Bub, Swd2 lost its occupancy through both ways on transcribed genes, but Set1 remains associated with the 5ʹ region of transcribed genes. Intriguingly, in the absence of Set1, the peak of Swd2 on the 5ʹ region was lost, but the peak on the 3ʹ region rather increased, suggesting that Set1 and CPF are competing with each other to obtain the common factor, Swd2, and Set1 is essential for redistributing Swd2 within transcribed genes, particularly to the 5ʹ region.

This study expands the Rad6-dependent chromatin occupancy of Swd2 confirmed by ChIP-qPCR results in a previous study to the genome levels [17]. Also, more importantly, this study newly shows that occupancy of Swd2 to transcribed gene is majorly through Set1 or CPF, and both ways are dependent on Rad6’s catalytic activity, and Set1 has a competitive relationship with CPF for Swd2. We believe that these data enhance the understanding of H2B ubiquitination-dependent H3K4me3 through Swd2/Cps35 and would be an important bridgehead to understand the relationship of two chromatin regulators (H2B ubiquitinase Rad6 and H3K4 methylase Set1) with transcriptional termination regulators (CPF).

## Results

### Determination of chromatin occupancy of Rad6, Swd2, and Set1 on transcribed genes

**Fig. 1:**
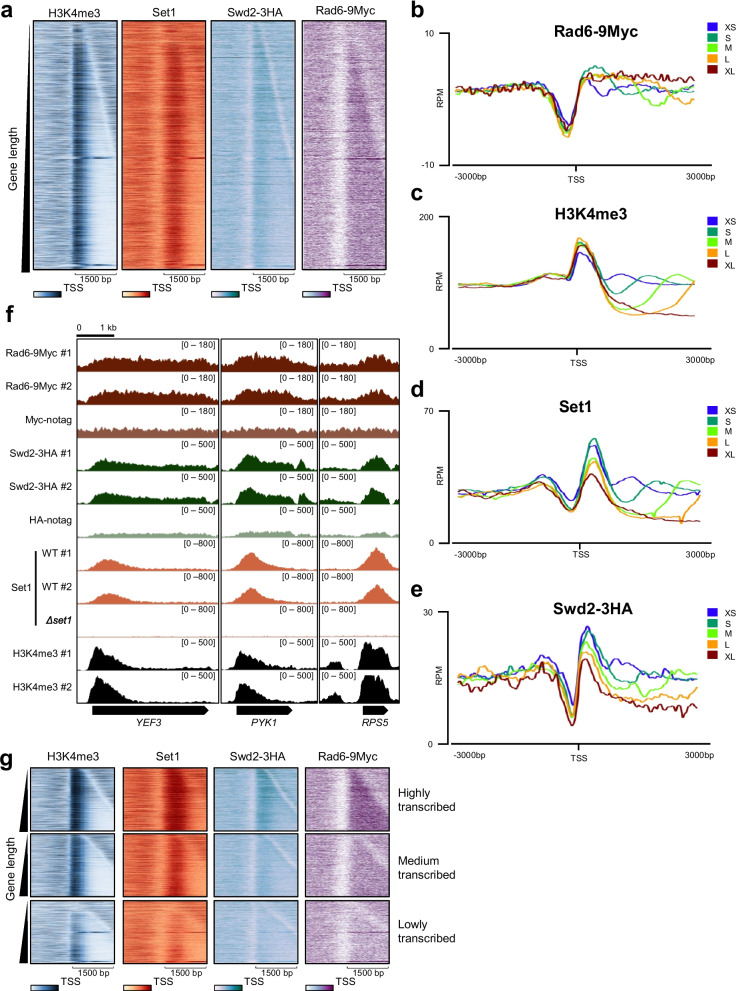
Determination of chromatin occupancy of Rad6, Swd2, and Set1 on transcribed genes. **a** The heatmaps represent the occupancy of H3K4me3, Set1, Swd2-3HA, and Rad6-9Myc in wild-type (WT) background around the TSS (± 1500 bp) of total protein coding genes (*n* = 6020). **b**–**e** Yeast total protein-coding genes are classified into five groups depending on the gene length; XS (< 750 bp), S (750 ~ 1500 bp), M (1500 ~ 2250 bp), L (2250 ~ 3000 bp), and XL (3000 ~ 3750 bp). The metagene shows the average patterns of chromatin occupancy of **b** Rad6-9Myc, **c** H3K4me3, **d** Set1, and **e** Swd2-3HA. *X*-axis: 6000-bp window around the transcription start sites (TSSs); *Y*-axis: reads per million (RPM). **f** The IGV tracks show the enrichments of Rad6-9Myc, Swd2-3HA, Set1, and H3K4me3 at three representative genes, *YEF3*, *PYK1*, and *RPS5*. **g** Yeast total protein-coding genes (*n* = 6020) are classified into three groups depending on reads per kilobase per million values of mRNA sequencing mapped to each gene. Number of genes of each group is 2006, 2006, and 2008 for high, medium, and low, respectively. Heatmaps show the occupancy of H3K4me3, Set1, Swd2-3HA, and Rad6-9Myc around the TSS (± 1500 bp) of highly transcribed genes (high), medium transcribed genes (medium), and lowly transcribed genes (low)

To improve our understanding of the correlation in transcribed gene occupancy between Rad6, Swd2, and Set1, we performed chromatin immunoprecipitation sequencing (ChIP-seq) for Rad6, Swd2, Set1, and H3K4me3. Epitope tags were added to the chromosomal copies of *RAD6* (Rad6-9Myc) or *SWD2* (Swd2-3HA) in yeast strains, and ChIP-seq was performed using antibodies against these tags. Additionally, ChIP-seq was performed with antibodies targeting Set1 and H3K4me3 in the wild-type strain. The distribution patterns of H3K4me3, Set1, Swd2, and Rad6 within the transcribed genes were obtained by sorting the yeast total genes (*n* = 6020) based on their gene length (Fig. 1a).

To determine the localization of Rad6, Swd2, Set1, and H3K4me3 within a gene, we adopted a previously used gene classification method where total protein-coding genes in yeast into five groups depending on their length: extra-small, small, medium, large, and extra-large [18]. Based on the average distribution of H2Bub and H3K4me3 in the five gene groups, H2Bub was suggested to be evenly distributed along the gene while H3K4me3 was present in pronounced peaks in proximity to transcription start sites (TSS) regions [18]. Accordingly, we found that H2B ubiquitinase Rad6 was also evenly distributed along a gene while H3K4me3 was present as a sharp peak near the TSS regions (Fig. 1b, c). Similar to H3K4me3, Set1 and Swd2 peaks were found near the TSS regions (Fig. 1d, e). We analyzed the ChIP-exo data for H2B and H2Bub from GSE147927 through our pipelines (Additional file 1: Fig. S1a–e) [19]. Coincident with the localization of Rad6, the heatmaps and metagenes show that H2Bub was evenly distributed along a gene (Additional file 1: Fig. S1a–e). These typical distributions of Rad6, Swd2, Set1, and H3K4me3 were also observed in integrative genomics viewer plots, highlighting the three representative genes (Fig. 1f).

To investigate the correlation between transcript levels and chromatin occupancy of Rad6, Swd2, Set1, and H3K4me3, we classified the total protein-coding genes in yeast (*n* = 6020) into high, medium, and low groups depending on the normalized transcript level of genes derived from mRNA-seq of the wild-type strain (data from GSE180992, [20]). As the transcript levels of genes increased, the chromatin occupancy of Rad6, Swd2, Set1, and H3K4me3 increased proportionally (Fig. 1g). Taken together, we confirmed that the transcription level of genes correlated with the chromatin occupancy of Rad6, Swd2, Set1, and H3K4me3. Furthermore, our results align with previous results, demonstrating that Rad6 and H2Bub are uniformly distributed along the gene [18]. In contrast, Set1 and Swd2 exhibited high occupancy in regions near the TSSs similarly to H3K4me3.

### Significant levels of Set1 occupy the 5′ region of transcribed genes in the absence of *RAD6*

**Fig. 2:**
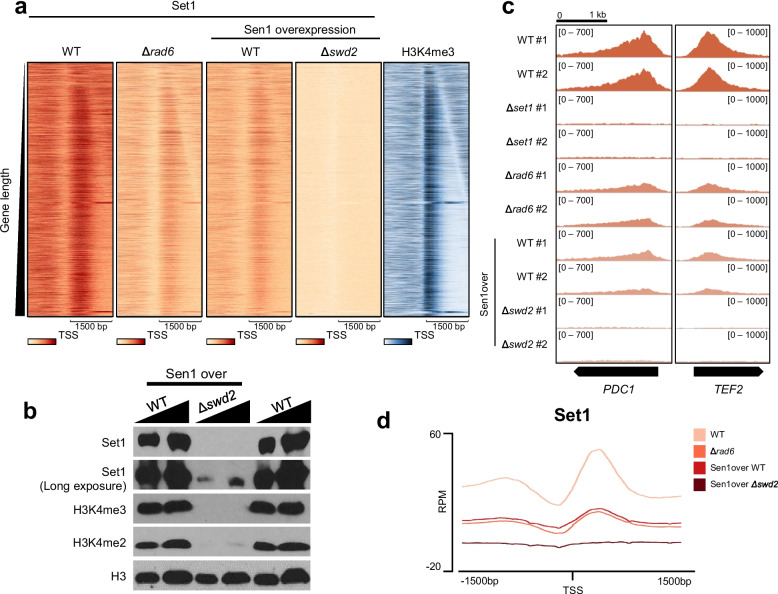
Significant Set1 occupies the 5ʹ region of transcribed genes in the absence of *RAD6*. **a** The heatmaps represent the occupancy of Set1 in wild-type (WT), Δ*rad6*, Sen1over WT, and Sen1over Δ*swd2*, and H3K4me3 of wild-type strain around the TSS (± 1500 bp) of total protein coding genes (*n* = 6020). **b** The levels of Set1, H3K4me3, H3K4me2, and H3 in the Sen1over WT, Sen1over Δ*swd2*, and wild-type strains were measured by western blotting. **c** The IGV tracks show the enrichments of Set1 at two representative genes, *PDC1* and *TEF2*, in WT, Δ*set1*, Δ*rad6*, Sen1over WT and Sen1over Δ*swd2* strains. **d** The metagenes represent the average distribution of Set1 near TSS regions (± 1500 bp) of total protein-coding genes (*n* = 6020) in WT, Δ*rad6*, Sen1over WT, and Sen1over Δ*swd2* strains

To identify how Rad6 and Swd2 affect the Set1 occupancy on transcribed genes, we performed Set1 ChIP-seq in *∆rad6* and *∆swd2* strains. To remove the background signals of the Set1 ChIP-seq, we also performed ChIP-seq in the *∆set1* strain. Consequently, we confirmed that deleting *RAD6* reduced the occupancy of Set1 on transcribed genes at genome-wide levels, although enrichment was still observed near the 5ʹ region of genes (Fig. 2a, c, d). To investigate whether reduction of Set1 occupancy in the *∆rad6* strain occurs with every yeast gene, we quantified the mapped reads per kilobase per million (RPKM) values of Set1 near the 5ʹ region of genes (− 100 to + 300 bp of TSS) in both wild-type and *∆rad6* strains and displayed this as a scatter plot (Additional file 1: Fig. S2a). If Set1 occupancy near the 5ʹ region of a gene in the *∆rad6* strain is similar to that of wild type, the dot is located near the *y* = *x* line (Additional file 1: Fig. S2a). Set1 occupancy at most genes was reduced in the *∆rad6* strain. Although several genes retained similar Set1 occupancy in the wild-type strain, their length was too short to assess the Set1 occupancy compared with Set1 levels near the 5ʹ region of genes. Therefore, Set1 occupancy is globally reduced at most *S. cerevisiae* genes in the *∆rad6* strain.

Although *SWD2* is an essential gene, the deletion strain of *SWD2* could be generated through overexpressing the truncated *SEN1* fragment [9]. Therefore, we performed Set1 ChIP-seq in Sen1-overexpressed wild-type (Sen1over WT) and *∆swd2* (Sen1over *∆swd2*) strains. Subsequently, the occupancy of Set1 on transcribed genes disappeared in the *∆swd2* strain, which may have been caused by the severely reduced levels of Set1 protein [9] (Fig. 2a, b). Interestingly, we observed that the Set1 occupancy on transcribed genes in the Sen1over WT strain decreased to the same levels as that in the *∆rad6* strain, whereas the levels of Set1 protein and H3K4 di- and tri-methylation were similar to that of the wild-type strain (Fig. 2a–d). This western blot data agreed with the results of a previous study where the protein levels of Set1 were unaffected by overexpression of the *SEN1* fragment [9].

To identify how the occupancy of Set1 in the 5ʹ region of genes is regulated by Sen1, we analyzed the Sen1 ChIP-exo data from GSE147927 (Additional file 1: Fig. S2b, c) [19]. In the heatmap, genes are sorted by gene length to more precisely confirm the localization of proteins on the transcribed genes (Additional file 1: Fig. S2b). The binding of Sen1 was detected across all transcribed genes with higher occupancy near the TSSs with a similar pattern to that of Set1, although the occupancy of Sen1 was closer to TSSs than that of Set1 (Additional file 1: Fig. S2b, c). Although how the overexpressed fragment of Sen1 interfered with the occupancy of Set1 on transcribed genes remained unclear, the occupancy levels of Set1 on transcribed genes in Sen1over WT strain was sufficient for significant cellular H3K4me3 and comparable with that of the *∆rad6* strain. These data suggest that in the absence of *RAD6*, Set1 occupancy is reduced to some extent but significant Set1 still occupies the 5ʹ region of genes.

### Catalytic activity of Rad6 is essential for the occupancy of Swd2 on transcribed genes

**Fig. 3:**
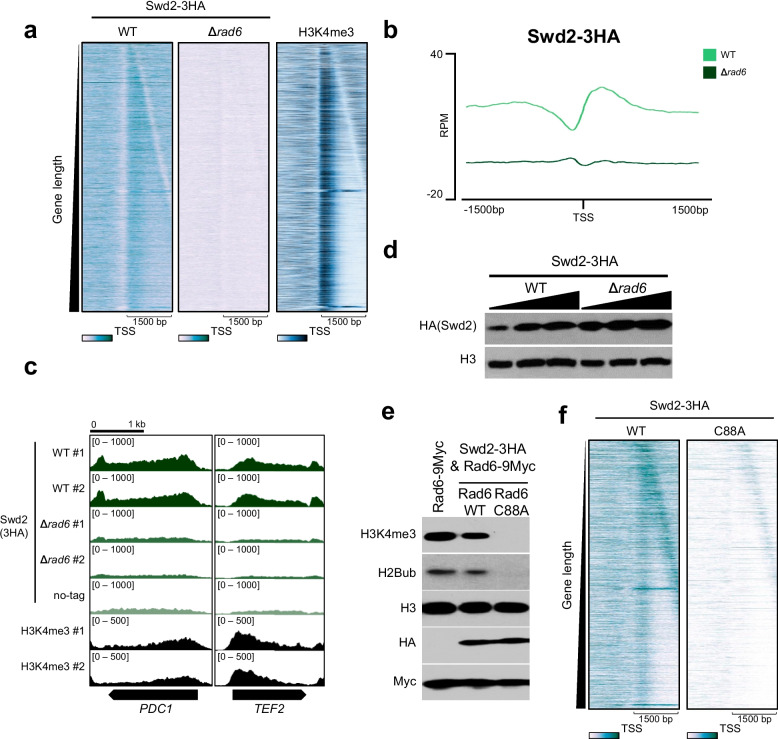
Catalytic activity of Rad6 is essential for the occupancy of Swd2 on transcribed genes. **a** The heatmaps represent the occupancy of Swd2-3HA in wild-type (WT) or ∆*rad6* background and H3K4me3 around the TSS (± 1500 bp) of total protein coding genes (*n* = 6020). **b** The metagenes represent the average distribution of Swd2-3HA near TSS regions (± 1500 bp) of total protein coding genes (*n* = 6020) of WT and *∆rad6* strains. **c** The IGV tracks show the Swd2-3HA enrichments in WT or *∆rad6* background and H3K4me3 at two representative genes, *PDC1* and *TEF2*. **d** The protein levels of Swd2-3HA in *SWD2-3HA* WT and *SWD2-3HA ∆rad6* were measured by western blotting. **e** The levels of H3K4me3, H2Bub, H3, Myc (Rad6-9Myc), and HA (Swd2-3HA) in the Rad6-9Myc WT, Swd2-3HA and Rad6-9Myc, and Swd2-3HA and Rad6-C88A-9Myc strains were measured by western blotting. **f** The heatmaps represent the occupancy of Swd2-3HA in wild-type (WT) or Rad6-C88A background around the TSSs (± 1500 bp) of total protein-coding genes (*n* = 6020)

To identify H2Bub-dependent chromatin occupancy of Swd2 at the genome-wide level, we added a *3HA* tag to a chromosomal copy of *SWD2* in wild-type or *∆rad6* background strains and conducted ChIP-seq with an anti-HA antibody (Fig. 3a–c); we also performed ChIP-seq in the untagged strain to remove the background signals of the HA tag ChIP-seq. We sorted the total protein-coding genes in yeast by gene length and compared the binding pattern with that of H3K4me3 (Fig. 3a). In the *∆rad6* strain, although the protein level of Swd2-3HA was not decreased, the occupancy of Swd2 on transcribed genes was significantly reduced at most protein-coding genes (Fig. 3a–d). The reduced chromatin binding of Swd2 in the *∆rad6* strain was comparable with that of the background signals in the untagged strain, indicating the removal of Swd2 binding upon *RAD6* deletion (Fig. 3a–c).

To identify whether the catalytic activity of Rad6 is important for the occupancy of Swd2 on transcribed genes, we chromosomally tagged Swd2 (*SWD2-3HA*) in the Rad6-9Myc wild-type and catalytically inactive Rad6-C88A-9Myc strains (Fig. 3e). Although H2Bub and H3K4me3 disappeared, the protein levels of Swd2-3HA remained unchanged in the Rad6-C88A strain (Fig. 3e). We compared the Swd2-3HA ChIP-seq in the Rad6-C88A strain with that of isogenic wild-type strain, and the results show that the occupancy of Swd2 on transcribed genes was severely reduced in the Rad6 catalytic dead mutant (Fig. 3f).

The levels of H2Bub and H3K4me3 were reduced by the addition of the 3HA tag at the C-terminus of *SWD2* (*SWD2-3HA*), and epitope tagging of Swd2 would therefore induce defects in the function of Swd2 to some extents (Fig. 3e). Nevertheless, considering that significant amount of H2Bub and H3K4me3 still remained, and the catalytic dead mutant of Rad6 still possesses the 3HA tagged Swd2, we consider that the impairment of Swd2 binding in the Rad6-C88A mutant results from the loss of the Rad6’s catalytic activity. Overall, the transcribed gene occupancy of Swd2 was completely dependent on Rad6 and its catalytic activity at most protein-coding genes. However, a significant amount of Set1 proteins could already occupy transcribed genes in the absence of Rad6, though this occupancy was enhanced by Rad6.

### Set1 redistributes Swd2 within transcribed genes to the 5′ region

**Fig. 4:**
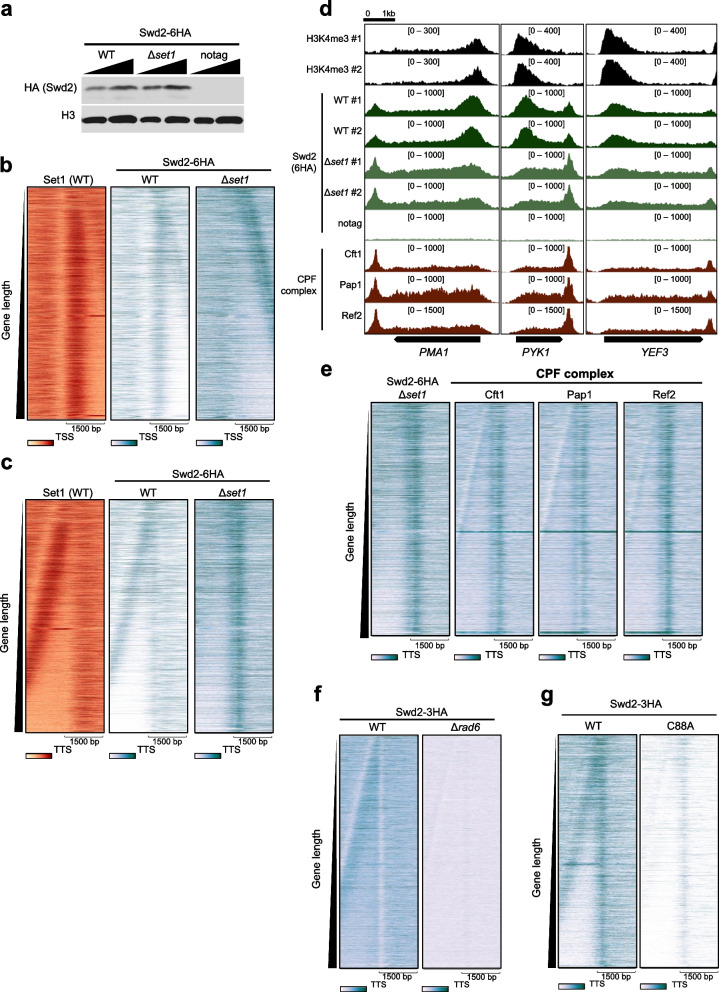
Set1 redistributes Swd2 within transcribed genes to the 5′ region. **a** The protein levels of Swd2-6HA in *SWD2-6HA* WT, *SWD2-6HA ∆set1*, and WT (notag) were measured by western blotting. The heatmaps show the occupancy of Swd2-6HA at 3000-bp window near **b** the transcription start sites (TSSs) or **c** the transcription termination sites (TTSs) in WT or Δ*set1* condition. **d** The IGV tracks show the enrichments of Swd2-6HA in WT or Δ*set1* background, and the three components of CPF, Cft1, Pap1, and Ref2 at three representative genes, *PMA1*, *PYK1*, and *YEF3*. The ChIP-exo data of Cft1, Pap1, and Ref2 have been obtained from GSE147927 and processed by our pipeline. **e** The heatmaps show the occupancy of Swd2-6HA in Δ*set1* strain, and three components of CPF, Cft1, Pap1, and Ref2 in wild-type strain around the transcription termination sites (± 1500 bp of TTSs). **f** The heatmaps represent the occupancy of Swd2-3HA in WT or Δ*rad6* background around the transcription termination site (± 1500 bp of TTSs) of total protein-coding genes (*n* = 6020). **g** The heatmaps represent the occupancy of Swd2-3HA in WT or Rad6-C88A background around the TTSs (± 1500 bp) of total protein-coding genes (*n* = 6020)

To identify whether Set1 is required for Swd2 occupancy, we performed HA ChIP-seq in yeast strains containing chromosomal *SWD2-6HA* in the wild-type and *∆set1* backgrounds. The protein levels of Swd2-6HA were unchanged by the deletion of *SET1* (Fig. 4a). The heatmaps show that peaks of Swd2 near TSSs were uniformly located from TSSs independently of the gene length in the wild-type condition (Fig. 4b). By contrast, in the *∆set1* strain, the Swd2 peaks near the TSSs decreased, but the occupancy of Swd2 increased at both of the other regions within transcribed genes and specifically at the transcription termination sites (TTSs) (Fig. 4b, c). In addition to being a component of Set1/COMPASS, Swd2 is a component of CPF transcription termination factors [36, 37]. Therefore, we also examined the occupancy of Swd2 near TTSs (Fig. 4c). To identify whether the occupancy of Swd2 in *∆set1* strain was similar to that of CPF complex components, we analyzed the ChIP-exo data of three components of CPF, Cft1, Pap1, and Ref2 from GSE147927 (Fig. 4d, e) [19]. The heatmaps illustrated that the global localization of Swd2 in the *∆set1* strain was similar to the occupancy of the three components of CPF (Fig. 4e and Additional file 1: Fig. S3). Notably, the occupancy of both Swd2 in the *∆set1* strain and the CPF occurred across the transcribed genes as well as near the TTSs. However, the occupancy of Swd2 across the transcribed genes and near the TTSs in both the *∆rad6* and Rad6-C88A strains disappeared (Fig. 4f, g).

## Discussion

H3K4me3 serves as a well-established hallmark indicative of active transcription, and numerous studies have elucidated the underlying molecular mechanisms associated with it. In the context of mammals, H3K4me3 plays a pivotal role in cell differentiation and development processes [4, 5]. In the case of differentiated cells, the H3K4me3 domain at the promoters of lineage determination factors is required to maintain cell identity [21]. A recent research endeavor has shed light on the involvement of H3K4me3 in global transcription regulation through RNA polymerase II pause-release [6]. Furthermore, it is well-documented that H3K4me3 is dependent on H2Bub in various organisms including yeast, fruit flies, and mammals [13, 14, 16, 22]. Therefore, unraveling the mechanistic interplay between H2Bub and H3K4me3 via Set1/COMPASS is of paramount importance in comprehending the evolutionary conservation of H3K4me3 [13, 14, 22, 23].

**Fig. 5:**
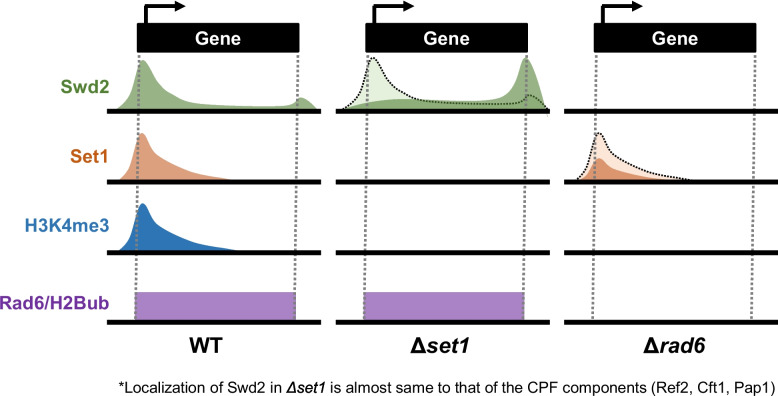
Summary: occupancy of Swd2 on transcribed genes is differentially regulated by Set1 and Rad6

Summary of this study is highlighted in Fig. 5. Swd2/Cps35 and its orthologs in *Drosophila* and mammals are required for the H2Bub-dependent H3K4me3 [17, 22, 23]. So, how the Swd2/Cps35 occupies the 5ʹ region of genes for H3K4me3 is an important issue for understanding the H2Bub-dependent H3K4me3. We reveal that two major Swd2 peaks exist near the 5ʹ region and 3ʹ region of genes. This study posits that Swd2 can establish its presence on transcribed genes through at least two mechanisms: (1) via the Set1/COMPASS and (2) through association with the CPF. It is noteworthy that the two pathways, owing to their close association with Swd2, are likely the most predominant pathways [7, 8, 12]. Our data support that catalytic activity of Rad6 is essential for all the peaks of Swd2 on the transcribed genes, that is, not only for the occupancy of Swd2 near the 5ʹ region, but also required for the Swd2 near the 3ʹ region. Based on the Swd2-6HA ChIP-seq data in the absence of the *SET1*, we realized that Set1 redistributes Swd2 to the 5ʹ region for accomplishments of its H3K4me3. Interestingly, the data suggest that Set1 competes with CPF to obtain quantitatively limited Swd2. Taken together, this study reveals that Rad6/H2Bub is essential for the occupancy of Swd2 on most genes in yeast, and Set1 redistributes Swd2 to the 5ʹ region of genes for H3K4me3 in the regions. Also, the results that catalytic activity of Rad6 is also required for the Swd2’s occupancy on the 3ʹ region and Set1 competes with CPF for Swd2 shed light on the potential regulation of CPF by Rad6 (or H2Bub) and Set1 (or H3K4me) through Swd2.

An interesting finding is that H2Bub by Rad6 would give authority to Set1 to lead the Swd2’s positioning to the 5ʹ region of genes. In the presence of H2Bub/Rad6, Set1 redistributes Swd2 to the 5ʹ region of genes (Fig. 4). However, in the absence of H2Bub/Rad6, while significant Set1 occupies the 5ʹ region of genes, these Set1 proteins did not contribute to the 5ʹ region positioning of Swd2 at all (Figs. 2 and 3). These data suggest that the Swd2 functions as a mediator translating the signal of H2Bub by Rad6 to the COMPASS. Two previous studies revealed the Swd2’s roles to mediate the H2Bub-dependent H3K4me3 [17, 24]. A study observed the loss of Swd2 from chromatin and the COMPASS purified from the H2Bub-deficient strains [17]. The other study revealed the H2Bub-dependent ubiquitination of Swd2, which is crucial for the H3K4me3 [24]. Based on these findings, a plausible model explaining how H2Bub promotes the Set1-dependent positioning of Swd2 to the 5ʹ region is that the binding of Swd2 to Set1 is enhanced, although the exact mechanisms remain to be identified. The direct modifications of Swd2 or other Set1 complexes and the involvement of the 3rd factor dependent on H2Bub would be the potential candidates.

Our data also unveil that Swd2 occupancy on the 3ʹ region of transcribed genes depends on the catalytic activity of Rad6 and is inhibited by Set1. An interesting finding is that both Set1 and Rad6 are required for the occupancy of Swd2 on the 5ʹ region but have the contrary roles for the occupancy on the 3ʹ region (Figs. 3 and 4). CPF-dependent Swd2 occupancy on the 3ʹ region is also dependent on H2Bub by Rad6, albeit further studies are warranted to ascertain whether the Rad6-mediated impact extends to the association of Swd2 with the CPF complex. Also, whether and how the H2Bub and H3K4 methylation affect the transcription termination by CPF through Swd2/Cps35 component should be identified through further studies. We believe that the data in this manuscript could be a bridgehead for studies regarding the relationship between a promoter-proximally positioned H3K4me3 and transcription termination by CPF.

Swd2 is an essential protein, although the deletion strain is viable in specific conditions. Interestingly, the lethality associated with *SWD2* can be suppressed by three distinct interventions: the deletion of *SET1* or *RAD6* and the overexpression of the *SEN1* fragment [9, 11]. A prior study hinted at the presence of an antagonistic relationship between the Set1/COMPASS and the CPF complex, both of which share an essential component Swd2 [11]. This study provided evidence that the three aforementioned conditions collectively attenuate the occupancy of Set1 upon transcribed genes. However, it remains an open question whether the reduced Set1 occupancy under these conditions serves as a common underlying mechanism for mitigating the lethality associated with *SWD2* deletion. Further research is needed to establish this causative link definitively.

## Conclusions

Our study revealed that two major Swd2 peaks exist near the 5ʹ region by Set1 and the 3ʹ region of genes which might be through the CPF. Our data support that the catalytic activity of Rad6 is essential for occupancy of Swd2 through both modes, and Set1 is required for the redistribution of Swd2 from the 3ʹ region to 5ʹ region, suggesting the competition between COMPASS and CPF for Swd2. This study is a bridgehead for the detailed understanding of H2Bub-dependent H3K4me3 and the potential functional relationship between H3K4 methylation by Set1 and transcription termination by CPF through its common factor Swd2/Cps35.

## Methods

### Strains

Strains used in this study are presented in Additional file 5: Table S1.

### Antibodies

The following antibodies were used in this study for the western blot and the ChIP assay: anti-H3K4me3 (Millipore Cat# 07–473, RRID: AB_1977252; 1/50,000 dilution for WB; 1 μl was added to a chromatin extract for ChIP), anti-H2Bub1 (Cell Signaling Technology Cat# 5546, RRID:AB_10693452; 1/2500 dilution for WB), anti-c-Myc (9E10) (Santa Cruz Biotechnology Cat# sc-40, RRID:AB_627268; 1/5000 dilution for WB; 10 μl was added to a chromatin extract for ChIP), and anti-HA (Santa Cruz Biotechnology Cat# sc-7392, RRID:AB_627809; 1/5000 dilution for WB; 2.5 μl was added to a chromatin extract for ChIP). Anti-H3 (1/100,000 dilution for WB), anti-H3K4me2 (1/50,000 dilution for WB), and anti-Set1(1/5000 dilution for WB; 2.5 μl was added to a chromatin extract for ChIP) were obtained from Shilatifard’s laboratory [15, 17].

### Western blot (WB)

Cell extract was prepared from overnight cultured cells in YPD (YP-dextrose) media at 30 °C. Harvested cells were homogenized by vortexing at 4 °C for 30 min. The lysates were boiled with loading dye and then analyzed for western blotting. Proceeding steps were performed as a previous study [17]. The western data in the main figures are the representative data of biologically duplicated results.

### Chromatin immunoprecipitation (ChIP)

ChIP assays were performed as described previously [17]. Briefly, cells were grown to the exponential phase (OD_600_ of 1.0) at 30 °C and harvested after cross-linking with formaldehyde for 5 min and quenching with 2.5 M glycine for 20 min. Cells were resuspended with FA-lysis buffer, and 0.5-mm glass beads were added. Bead beating was performed for 30 min at 4 °C, and the chromatin of lysed cells was sheared by sonication. Immunoprecipitation was performed with antibodies against Rad6-9Myc, Swd2-3HA, Swd2-6HA, Set1, and H3K4me3. In Rad6-9Myc, Swd2-3HA, Swd2-6HA, Set1, and H3K4me3 for ChIP experiments, 5% of chromatin extract of *Candida albicans ∆set1* strain (set1 null mutant (∆SETdU) from [25]; for Set1 ChIP-seq) or 10% of chromatin extract of *Schizosaccharomyces pombe* (strain 972 h- (ATCC 24843); for the other ChIP-seqs) was added for spike-in normalization. The A/G agarose (#sc-2003, Santa Cruz Biotechnology) was used for precipitating the DNA. After washing, reverse cross-linking and DNA precipitation were performed.

### ChIP-seq and analysis

ChIP-seq DNA samples were quantified by Quant-iT PicoGreen dsDNA Assay kit, and 10 ng of DNA was used for the starting material of library preparation. ChIP-Seq library was constructed with NEBNext® ChIP-Seq Library Prep Master Mix Set for Illumina® (#E6240, NEB) according to the manufacturer’s instructions. Sequencing was performed using Illumina HiSeq 2500 System (RRID:SCR_016383) by the manufacturer’s protocol, and 51-bp single-end reads were generated for each sample. The sequencing adapter removal and quality-based trimming were performed by Trimmomatic v.0.36 (RRID:SCR_011848) [26]. Cleaned reads were mapped to the reference genome using Bowtie 2 (RRID:SCR_016368) v.2.2.5 with the default parameter [27]. *S. pombe* reads were calculated and used for normalization by adjusting spike-in reads to one million reads for each sample. The resulted data were used for visualization by EaSeq [28] and Integrative Genomics Viewer (IGV) (RRID:SCR_011793) [29].

ChIP-seq for Rad6-9Myc, Swd2-3HA (WT, *∆rad6* or Rad6-C88A), Swd2-6HA (WT or *∆set1*), Set1 (WT, *∆set1*, *∆rad6*, Sen1over WT, or Sen1over  ∆*swd2*), and H3K4me3 were performed in biologically duplicate. We performed ChIP-seq using Myc or HA antibodies with a strain without any epitope tag (No-tag) once to consider the background signals by non-specific binding. IGV track data in this manuscript were drawn with the resulting data. Then, the Myc or HA signals to the No-tag control strain were substracted from each of the duplicate ChIP-seq data of Myc-tagged Rad6 (Rad6-9Myc) or HA-tagged Swd2 (Swd2-3HA or Swd2-6HA), respectively. Data supporting the reproducibility of the resulting duplicate ChIP-seq data were included in this paper with the Additional file2 (Data supporting the reproducibility of the ChIP-seq results). The average values of the duplicate data were used for the heatmaps and metagene plots in this manuscript.

Total protein-coding genes (*n* = 6020) were selected after excluding noncoding exons from 7589 genes of the sacCer3 reference genome (Uniprot, 07/15/2017 version). After calculating the mean RPM (reads per million) values of each gene, heatmaps and anchor plots were drawn through the guidance of EaSeq [28].

### Supplementary Information

**Additional file 1: Fig. S1.** Determination of H2B ubiquitination on transcribed gene. **a** The heatmaps represent the occupancy of H2B and H2BK123 ubiquitination around the TSSs (Transcription start sites; ±1500bp) of total protein coding genes (*n* = 6020). **b-e** Metagenes show the average distribution of **b-c** H2B or **d-e** H2Bub around the TSS (±1,500bp) in WT strain. **Fig. S2.** Significant levels of Set1 occupy the 5ʹ region of transcribed genes in the absence of *RAD6*. **a** The scatter plot represents the normalized Set1 occupancy near TSSs (from -100bps to +300bps of TSSs) of total protein coding genes (*n* = 6020) in WT and Δ*rad6* strains. To calculate the normalized Set1 occupancy near TSSs, after The RPKM values of Set1 ChIP-seq mapped near TSSs (from -100bps to +300bps of TSSs) in WT, Δ*rad6* and Δ*set1* strains had been calculated, the values of Δ*set1* strains have been subtracted from the Wildtype and *∆rad6* strains. **b** The heatmaps represent the occupancy of Sen1 and Set1 around the TSSs (Transcription start sites; ±1500bp) of total protein coding genes (*n* = 6,020). **c** The IGV tracks show the enrichments of Sen1 in WT strain, and Set1 in WT, Δ*set1* and Sen1over WT strains at three representative genes, *PMA1*, *PYK1* and *YEF3*. **Fig. S3.** Set1 redistributes Swd2 within transcribed genes to the 5ʹ region. The heatmaps show the occupancy of Swd2-6HA in Δ*set1* strain, and three CPF complex components, Cft1, Pap1 and Ref2 in wildtype strain around the transcription start sites (±1500bp of TSSs).

**Additional file 2. **Data supporting the reproducibility of the ChIP-seq results.

**Additional file 3. **Uncropped blots for western blot data.

**Additional file 4. **Reads of spikes-in chromatin mapped to the genome of *C. albicans* (for Set1 ChIP-seq) or *S. pombe* (other ChIP-seqs).

**Additional file 5:** **Table S1.** Strains used in this study.

## Data Availability

All data generated or analyzed during this study are included in this published article and its supplementary information files (Additional files 1, 2, 3 and 4) and publicly available repositories. The yeast strains generated in this study are included in Additional file 5 and available from the corresponding authors upon request. Sequencing datasets are available in the NCBI Gene Expression Omnibus (GEO; https://www.ncbi.nlm.nih.gov/geo/). Swd2-3HA(WT, *∆rad6*, Rad6-C88A), Rad6-9Myc (WT, Rad6-C88A), and Swd2-6HA(WT, *∆set1*) ChIP-seq datasets: accession number GSE193209 (https://www.ncbi.nlm.nih.gov/geo/query/acc.cgi?acc=GSE193209). Set1 ChIP-seq datasets in WT, *∆set1*, *∆rad6*, Sen1overexpressed WT, and *∆swd2* strains: accession number GSE234564 (https://www.ncbi.nlm.nih.gov/geo/query/acc.cgi?acc=GSE234564). H3K4me3 ChIP-seq datasets containing the wild-type strain: GSE261160 (https://www.ncbi.nlm.nih.gov/geo/query/acc.cgi?acc=GSE261160). ChIP-exo data for H2B, H2Bub, Sen1, Cft1, Pap1, and Ref2 from GSE147927 (https://www.ncbi.nlm.nih.gov/geo/query/acc.cgi?acc=GSE147927) [19]. mRNA-seq data of the wild-type strain from GSE180992 (https://www.ncbi.nlm.nih.gov/geo/query/acc.cgi?acc=GSE180992) [20].

## Abbreviations

H3K4me3: Histone H3K4 tri-methylation
H2Bub: Histone H2B K123 mono-ubiquitination
COMPASS: Complex of proteins associated with Set1
CPF: Cleavage and polyadenylation factor
ChIP: Chromatin immunoprecipitation
qPCR: Quantitative PCR
ChIP-seq: Chromatin immunoprecipitation sequencing
TSS: Transcription start site
RPKM: Reads per kilobase per million
HA: Hemagglutinin
TTS: Transcription termination site
