# Supplementary material for: Swd2/Cps35 determines H3K4 tri-methylation via interactions with Set1 and Rad6
Source: BMC Biol. 2024 May 3;22:105. doi: 10.1186/s12915-024-01903-3 (PMC11069235; doi:10.1186/s12915-024-01903-3)
Supplement: Supplementary file 1 — Additional file 1: Fig. S1. Determination of H2B ubiquitination on transcribed gene. a The heatmaps represent the occupancy of H2B and H2BK123 ubiquitination around the TSSs (Transcription start sites; ±1500bp) of total protein coding genes (n = 6020). b-e Metagenes show the average distribution of b-c H2B or d-e H2Bub around the TSS (±1,500bp) in WT strain. Fig. S2. Significant levels of Set1 occupy the 5ʹ region of transcribed genes in the absence of RAD6. a The scatter plot represents the normalized Set1 occupancy near TSSs (from -100bps to +300bps of TSSs) of total protein coding genes (n = 6020) in WT and Δrad6 strains. To calculate the normalized Set1 occupancy near TSSs, after The RPKM values of Set1 ChIP-seq mapped near TSSs (from -100bps to +300bps of TSSs) in WT, Δrad6 and Δset1 strains had been calculated, the values of Δset1 strains have been subtracted from the Wildtype and ∆rad6 strains. b The heatmaps represent the occupancy of Sen1 and Set1 around the TSSs (Transcription start sites; ±1500bp) of total protein coding genes (n = 6,020). c The IGV tracks show the enrichments of Sen1 in WT strain, and Set1 in WT, Δset1 and Sen1over WT strains at three representative genes, PMA1, PYK1 and YEF3. Fig. S3. Set1 redistributes Swd2 within transcribed genes to the 5ʹ region. The heatmaps show the occupancy of Swd2-6HA in Δset1 strain, and three CPF complex components, Cft1, Pap1 and Ref2 in wildtype strain around the transcription start sites (±1500bp of TSSs). [file 12915_2024_1903_MOESM1_ESM.pptx]

## Slide 1
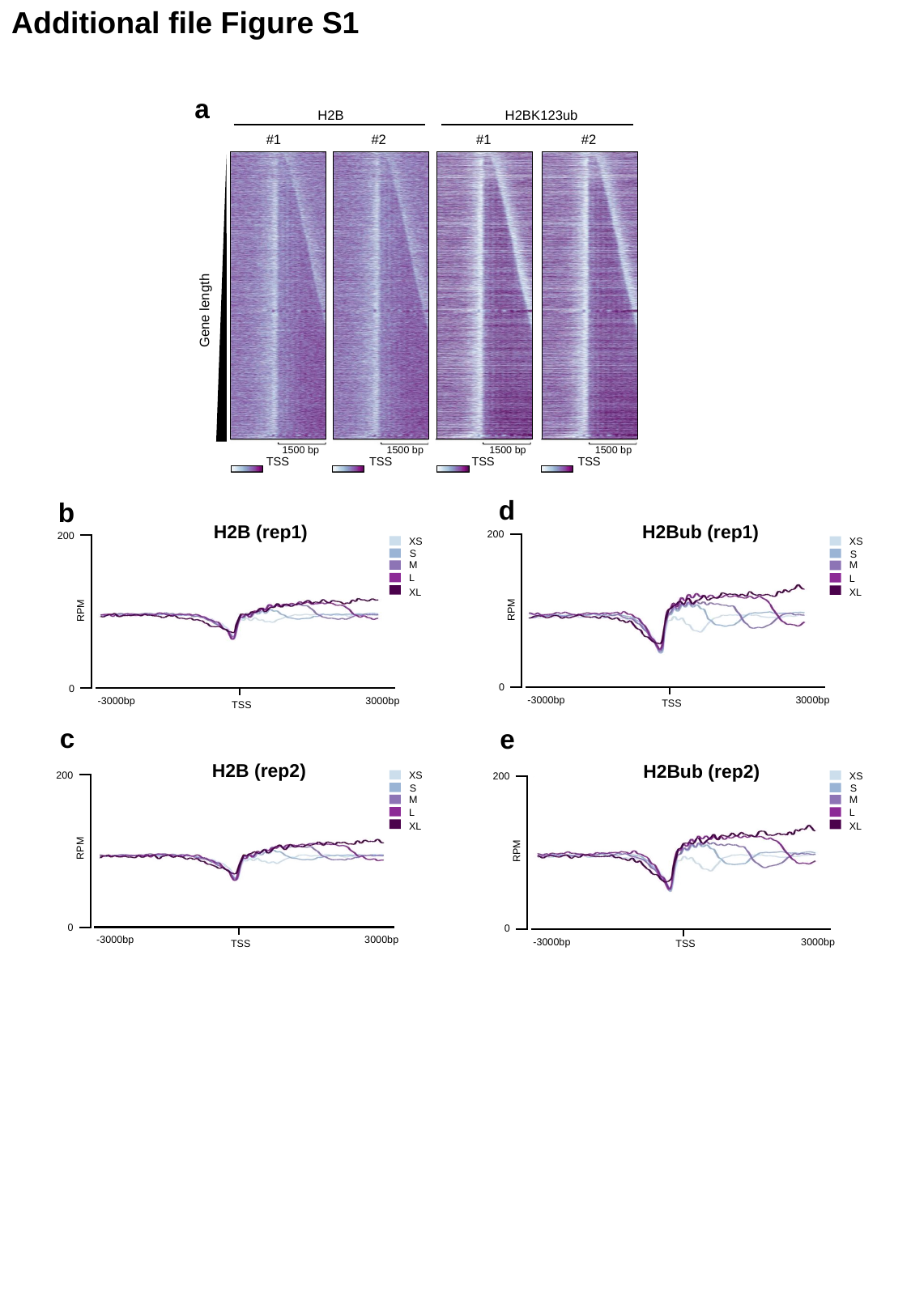

Additional file Figure S1
a
H2B
H2BK123ub
#1
#2
#1
#2
Gene length
1500 bp
1500 bp
1500 bp
1500 bp
TSS
TSS
TSS
TSS
d
b
H2B (rep1)
200
XS
S
M
L
XL
RPM
0
-3000bp
3000bp
TSS
H2Bub (rep1)
200
XS
S
M
L
XL
RPM
0
-3000bp
3000bp
TSS
c
e
H2B (rep2)
200
0
-3000bp
3000bp
TSS
XS
S
M
L
XL
RPM
H2Bub (rep2)
XS
200
S
M
L
XL
RPM
0
-3000bp
3000bp
TSS

## Slide 2
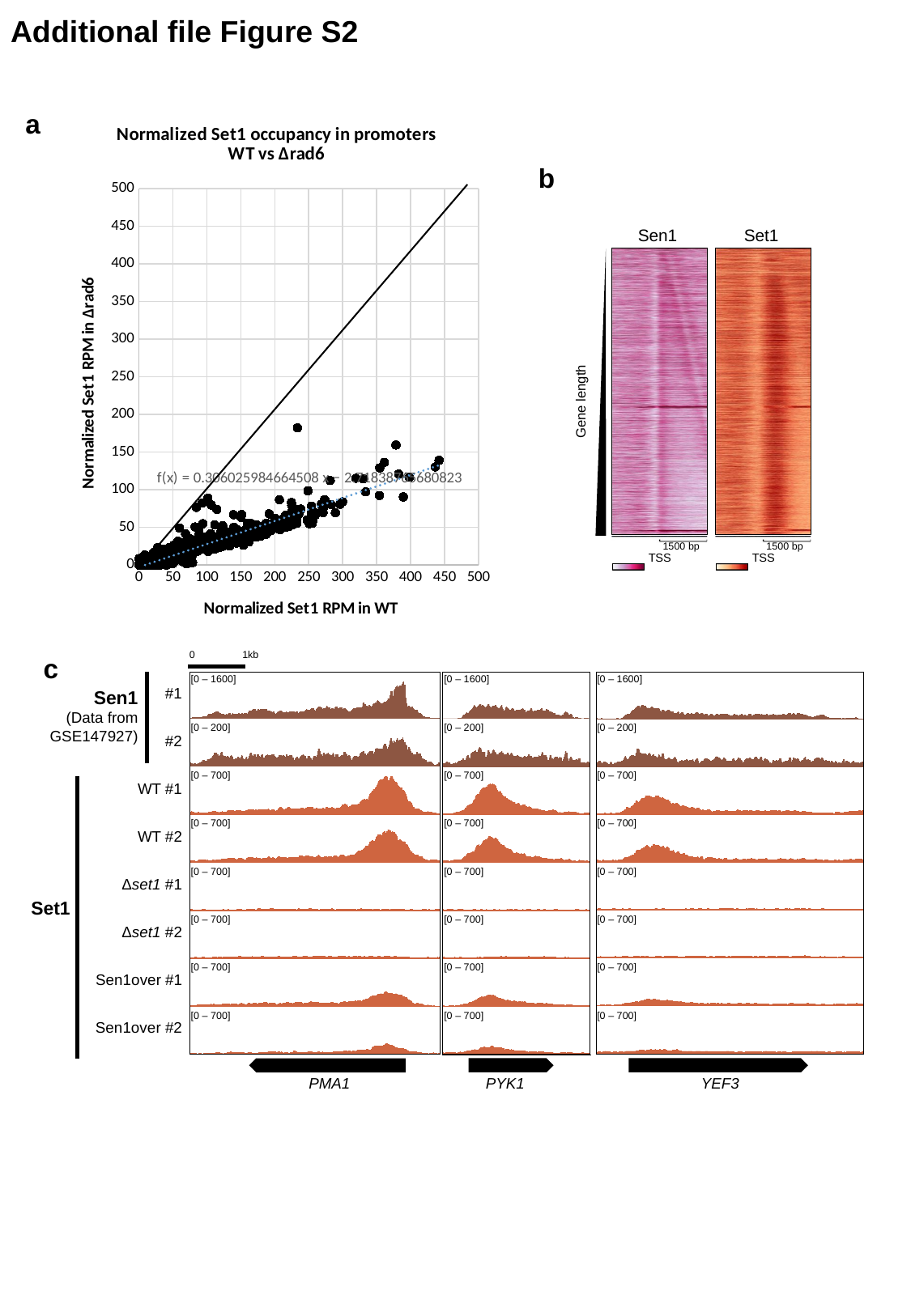

Additional file Figure S2
a
### Chart: Normalized Set1 occupancy in promoters
WT vs Δrad6
| Category | Normdrad6 |
|---|---|b
Sen1
Set1
Gene length
1500 bp
1500 bp
TSS
TSS
0
1kb
[0 – 1600]
[0 – 200]
[0 – 700]
[0 – 700]
[0 – 700]
[0 – 700]
[0 – 700]
[0 – 700]
[0 – 1600]
[0 – 200]
[0 – 700]
[0 – 700]
[0 – 700]
[0 – 700]
[0 – 700]
[0 – 700]
[0 – 1600]
[0 – 200]
[0 – 700]
[0 – 700]
[0 – 700]
[0 – 700]
[0 – 700]
[0 – 700]
#1
Sen1
(Data from
 GSE147927)
#2
WT #1
WT #2
Δset1 #1
Set1
Δset1 #2
Sen1over #1
Sen1over #2
PMA1
PYK1
YEF3
c

## Slide 3
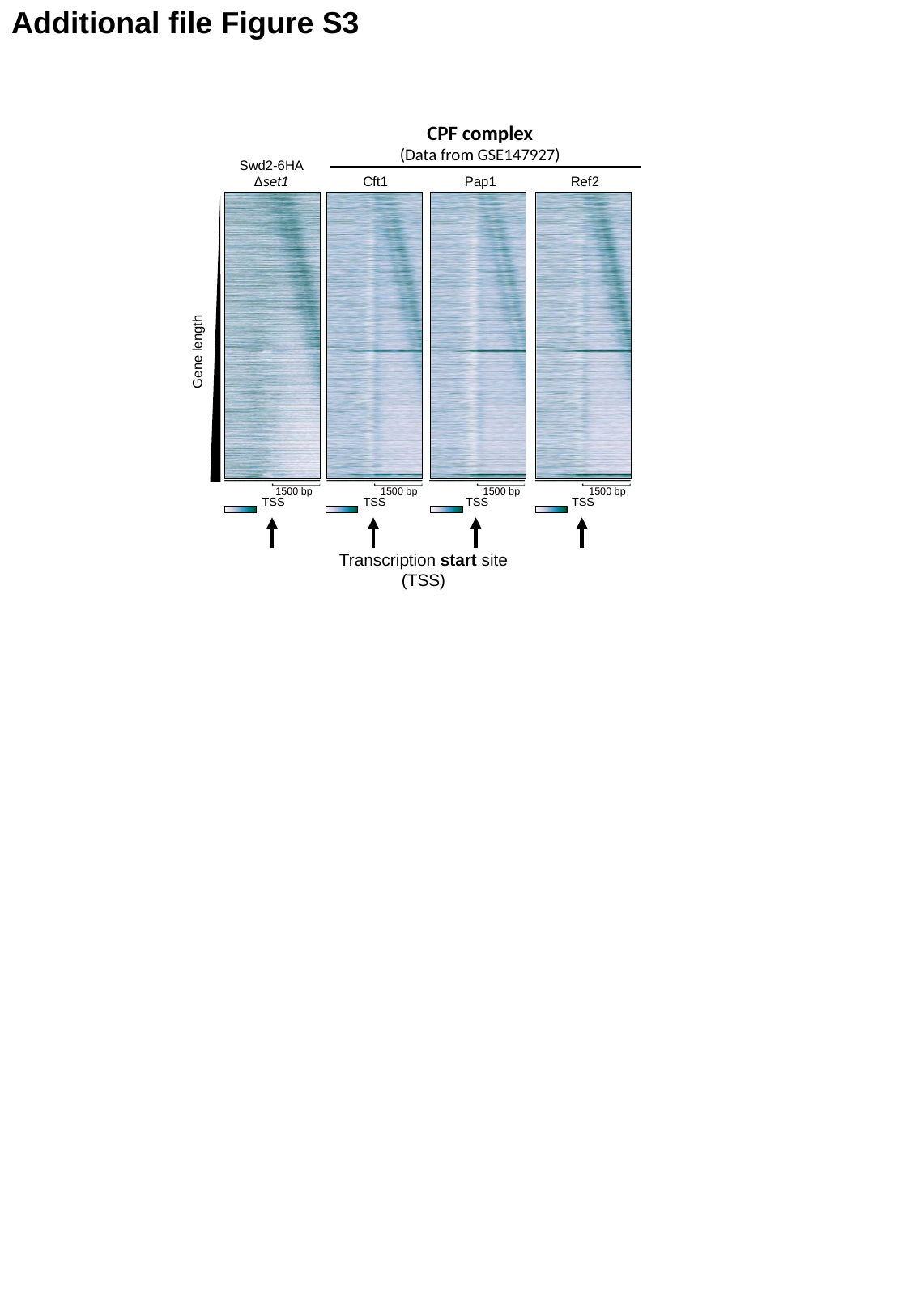

Additional file Figure S3
CPF complex
(Data from GSE147927)
Swd2-6HA Δset1
Cft1
Pap1
Ref2
Gene length
1500 bp
1500 bp
1500 bp
1500 bp
TSS
TSS
TSS
TSS
Transcription start site
(TSS)
