# Supplementary material for: Swd2/Cps35 determines H3K4 tri-methylation via interactions with Set1 and Rad6
Source: BMC Biol. 2024 May 3;22:105. doi: 10.1186/s12915-024-01903-3 (PMC11069235; doi:10.1186/s12915-024-01903-3)
Supplement: Supplementary file 2 — Additional file 2. Data supporting the reproducibility of the ChIP-seq results. [file 12915_2024_1903_MOESM2_ESM.pptx]

## Slide 1
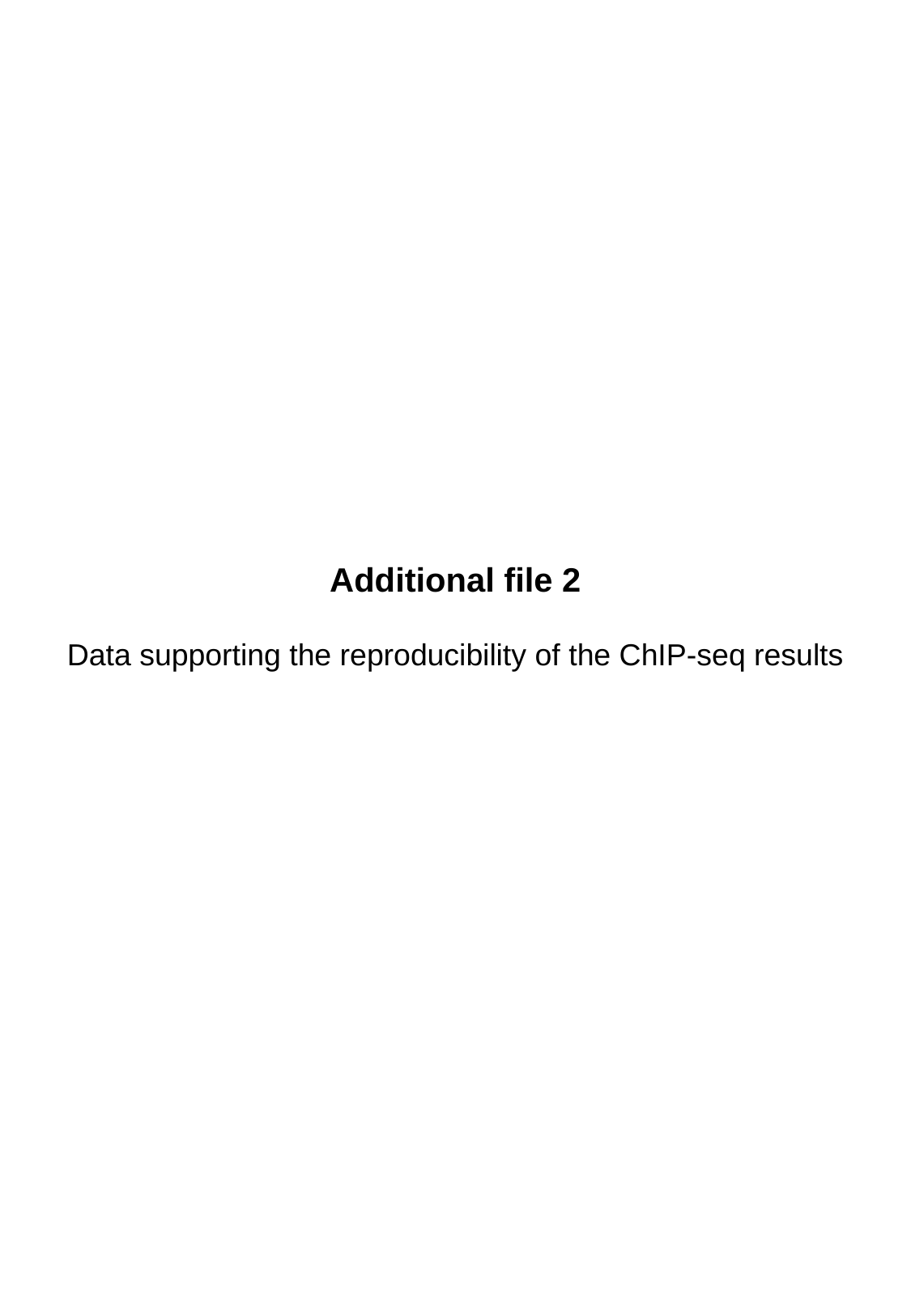

Additional file 2
Data supporting the reproducibility of the ChIP-seq results

## Slide 2
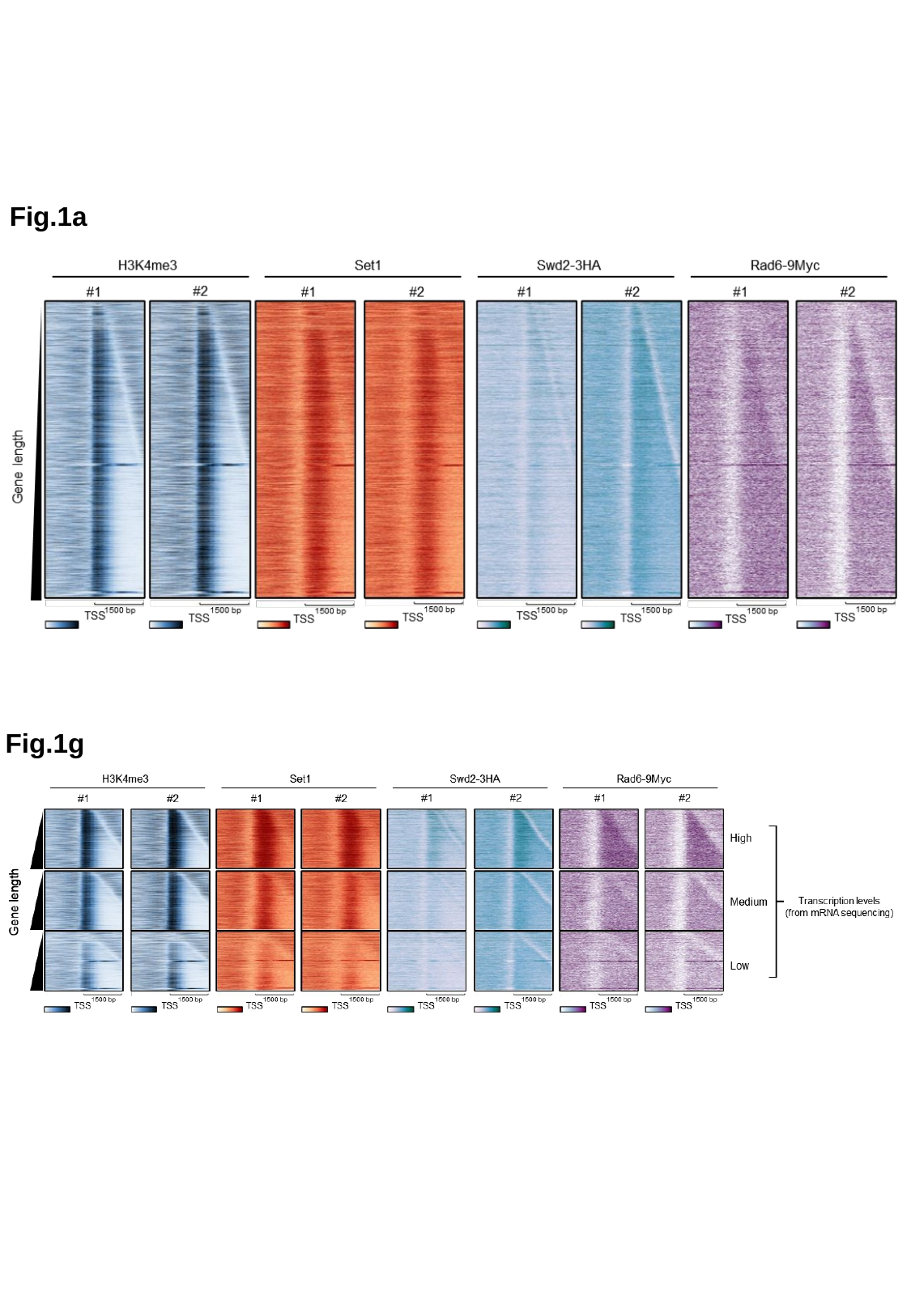

Fig.1a
Fig.1g

## Slide 3
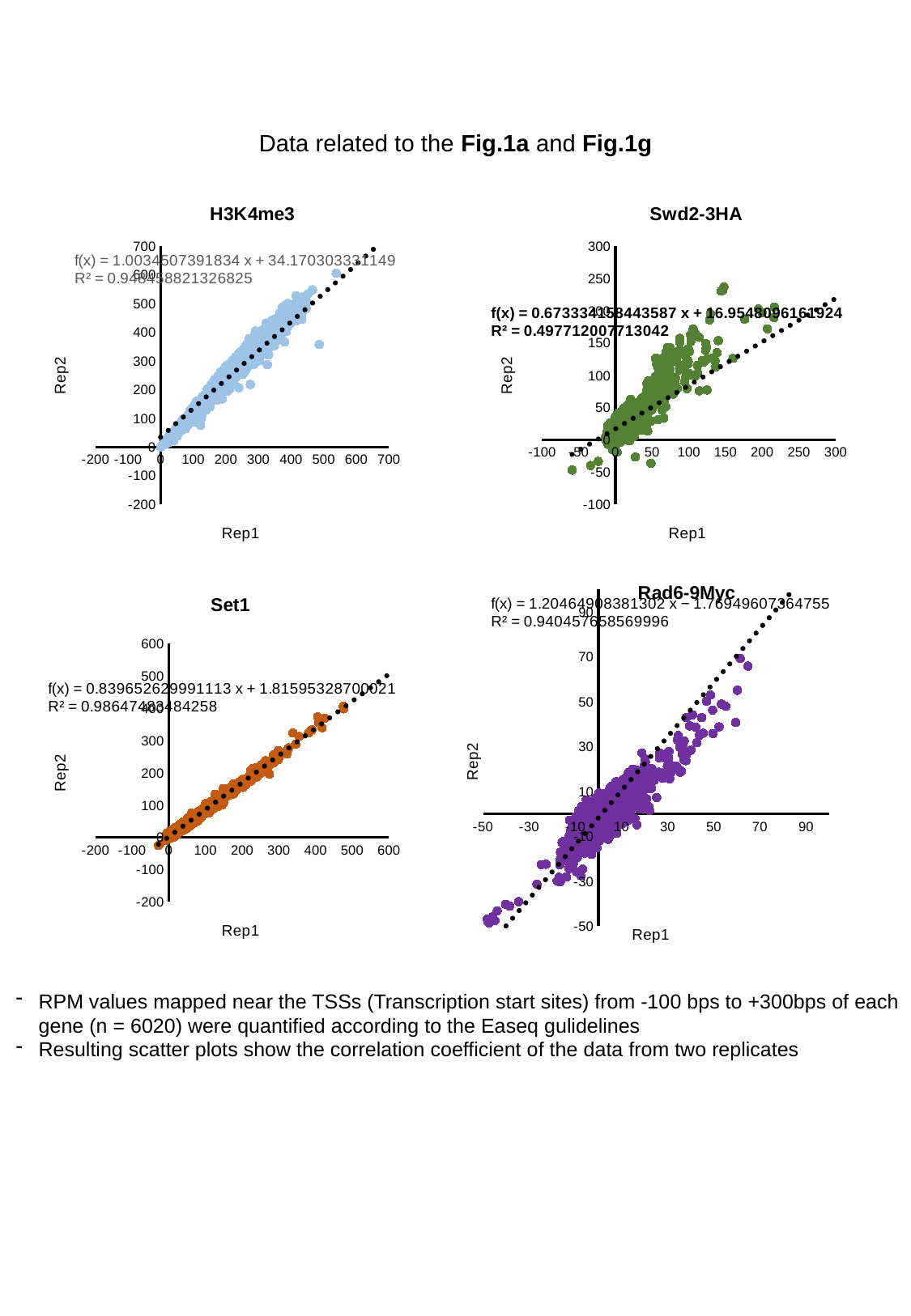

Data related to the Fig.1a and Fig.1g
### Chart: H3K4me3
| Category | 551WT-2 |
|---|---|
### Chart: Swd2-3HA
| Category | Swd2-3HA_minus_notag_2 |
|---|---|
### Chart: Rad6-9Myc
| Category | Rad6-9myc_minus_notag_2 |
|---|---|
### Chart: Set1
| Category | a-Set1_WT_minus_dset1_rep2 |
|---|---|RPM values mapped near the TSSs (Transcription start sites) from -100 bps to +300bps of each gene (n = 6020) were quantified according to the Easeq gulidelines
Resulting scatter plots show the correlation coefficient of the data from two replicates

## Slide 4
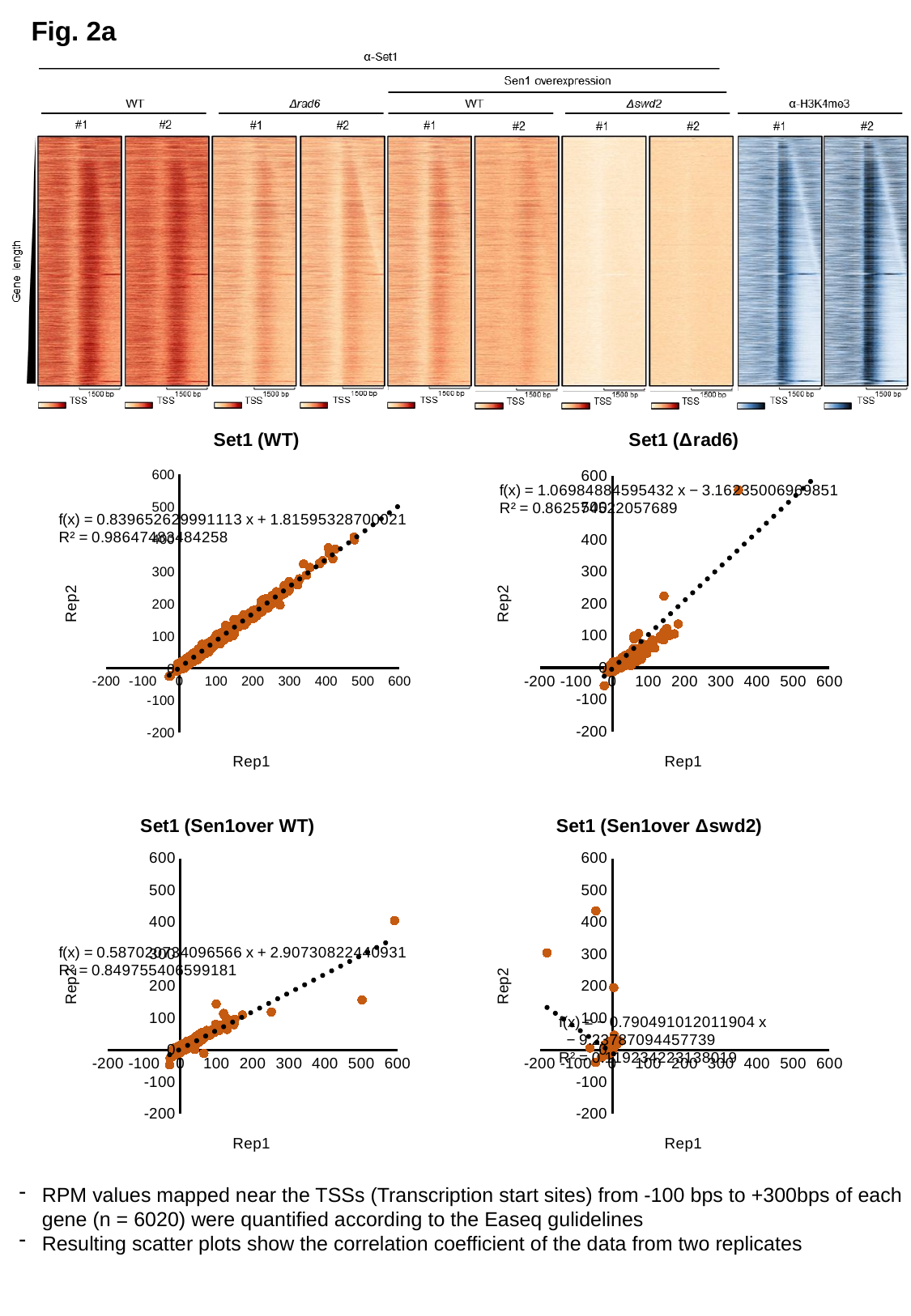

Fig. 2a
### Chart: Set1 (WT)
| Category | a-Set1_WT_minus_dset1_rep2 |
|---|---|
### Chart: Set1 (Δrad6)
| Category | a-Set1_drad6_minus_dset1_rep2 |
|---|---|
### Chart: Set1 (Sen1over WT)
| Category | a-Set1_Sen1overWT_minus_dset1_rep2 |
|---|---|
### Chart: Set1 (Sen1over Δswd2)
| Category | a-Set1_Sen1overdswd2_minus_dset1_rep2 |
|---|---|RPM values mapped near the TSSs (Transcription start sites) from -100 bps to +300bps of each gene (n = 6020) were quantified according to the Easeq gulidelines
Resulting scatter plots show the correlation coefficient of the data from two replicates

## Slide 5
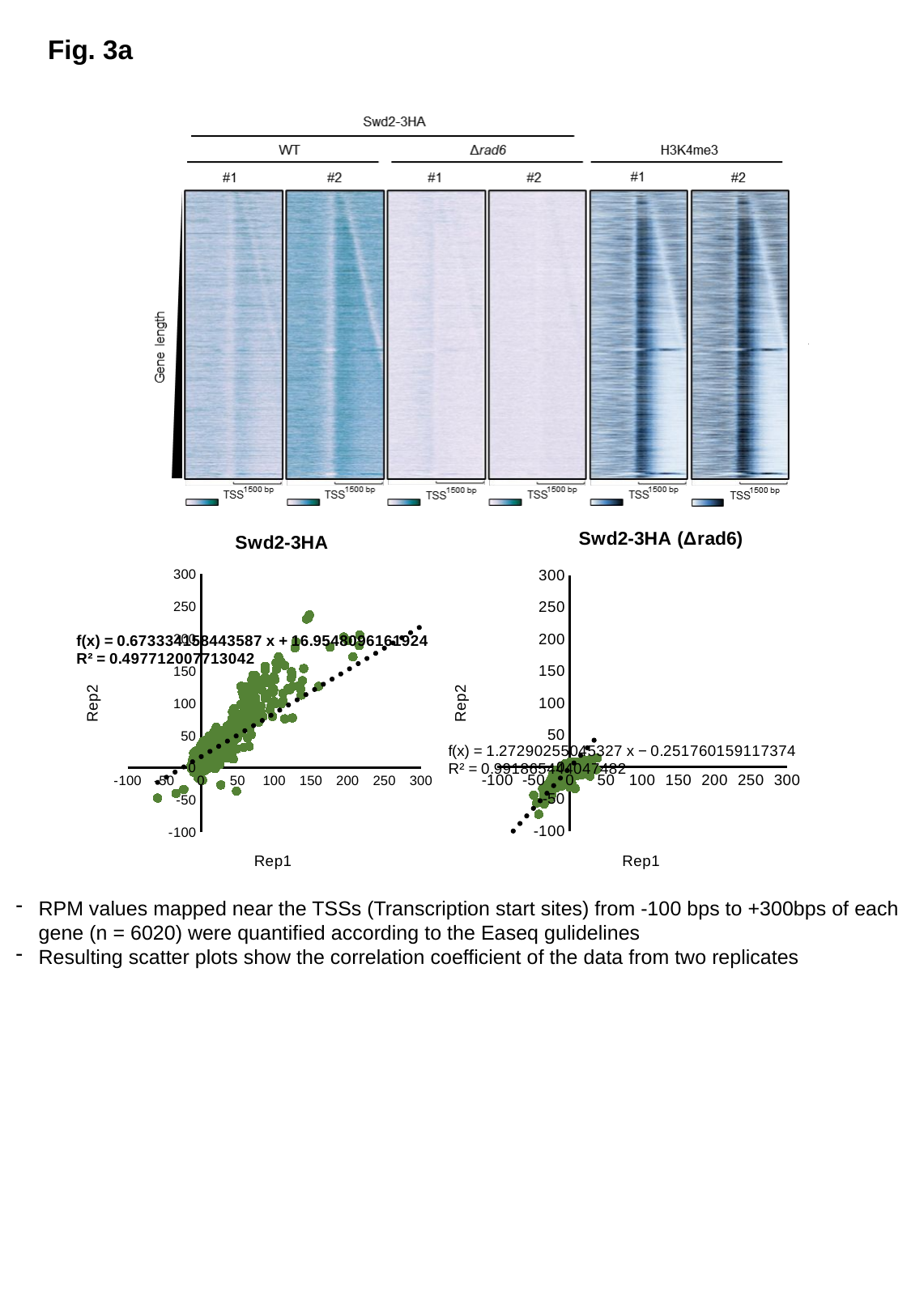

Fig. 3a
### Chart: Swd2-3HA
| Category | Swd2-3HA_minus_notag_2 |
|---|---|
### Chart: Swd2-3HA (Δrad6)
| Category | Swd2-3HA_drad6_minus_notag_2 |
|---|---|RPM values mapped near the TSSs (Transcription start sites) from -100 bps to +300bps of each gene (n = 6020) were quantified according to the Easeq gulidelines
Resulting scatter plots show the correlation coefficient of the data from two replicates

## Slide 6
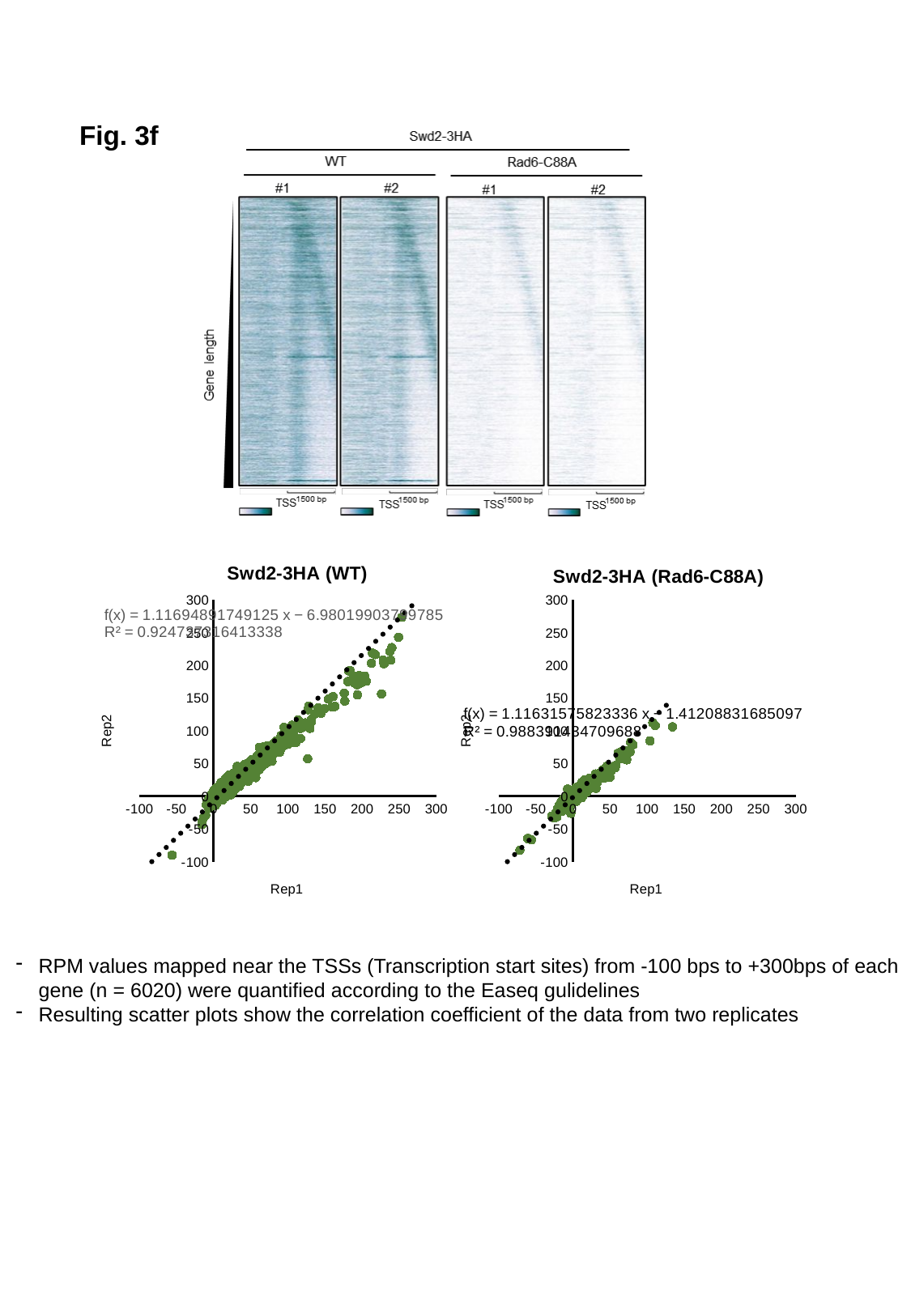

Fig. 3f
### Chart: Swd2-3HA (WT)
| Category | Swd2-3HA_Rad6_WT_minus_notag_2 |
|---|---|
### Chart: Swd2-3HA (Rad6-C88A)
| Category | Swd2-3HA_C88A_minus_notag_2 |
|---|---|RPM values mapped near the TSSs (Transcription start sites) from -100 bps to +300bps of each gene (n = 6020) were quantified according to the Easeq gulidelines
Resulting scatter plots show the correlation coefficient of the data from two replicates

## Slide 7
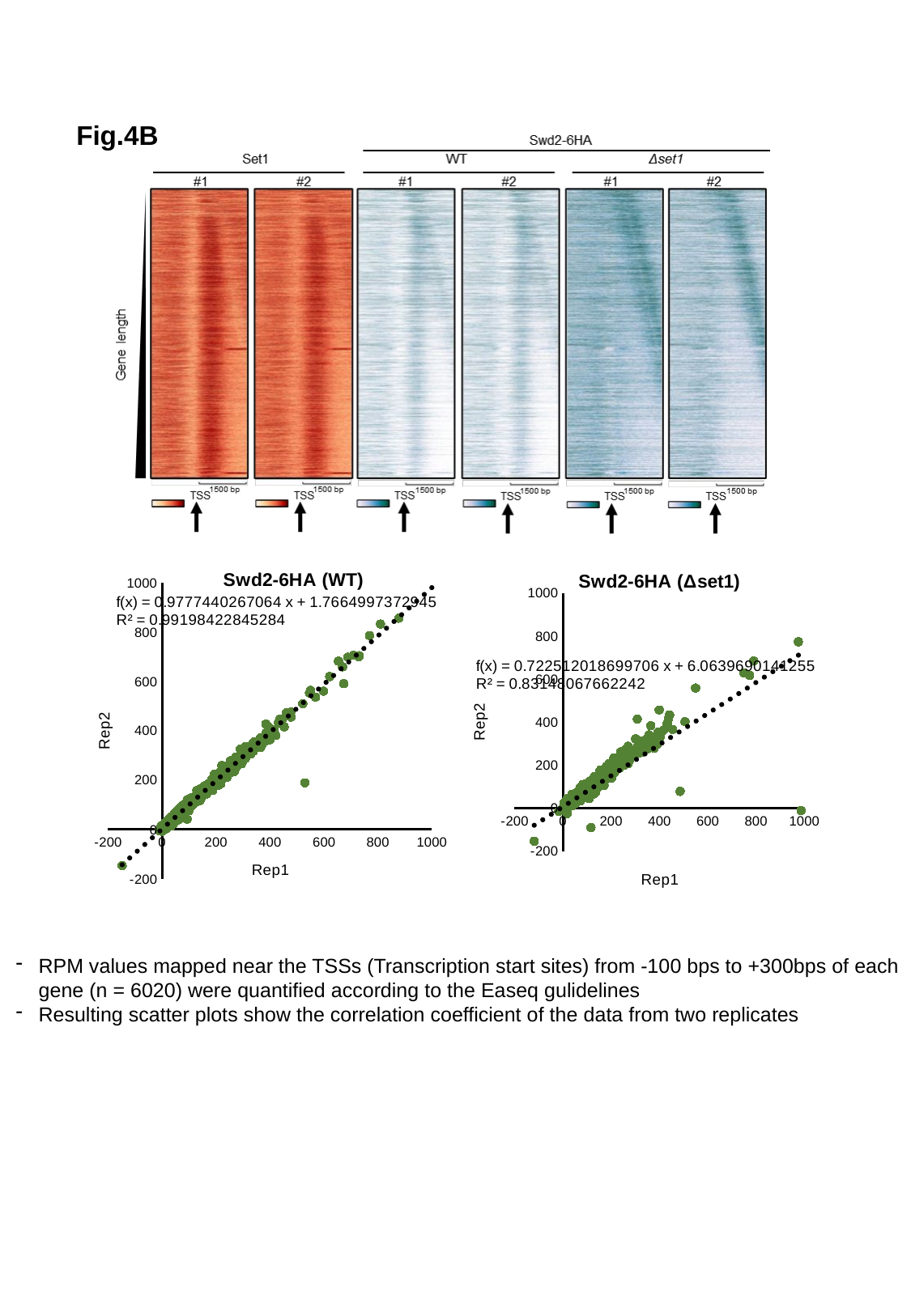

Fig.4B
### Chart: Swd2-6HA (Δset1)
| Category | Swd2-6HA_dset1_minus_notag2 |
|---|---|
### Chart: Swd2-6HA (WT)
| Category | Swd2-6HA_minus_notag2 |
|---|---|RPM values mapped near the TSSs (Transcription start sites) from -100 bps to +300bps of each gene (n = 6020) were quantified according to the Easeq gulidelines
Resulting scatter plots show the correlation coefficient of the data from two replicates

## Slide 8
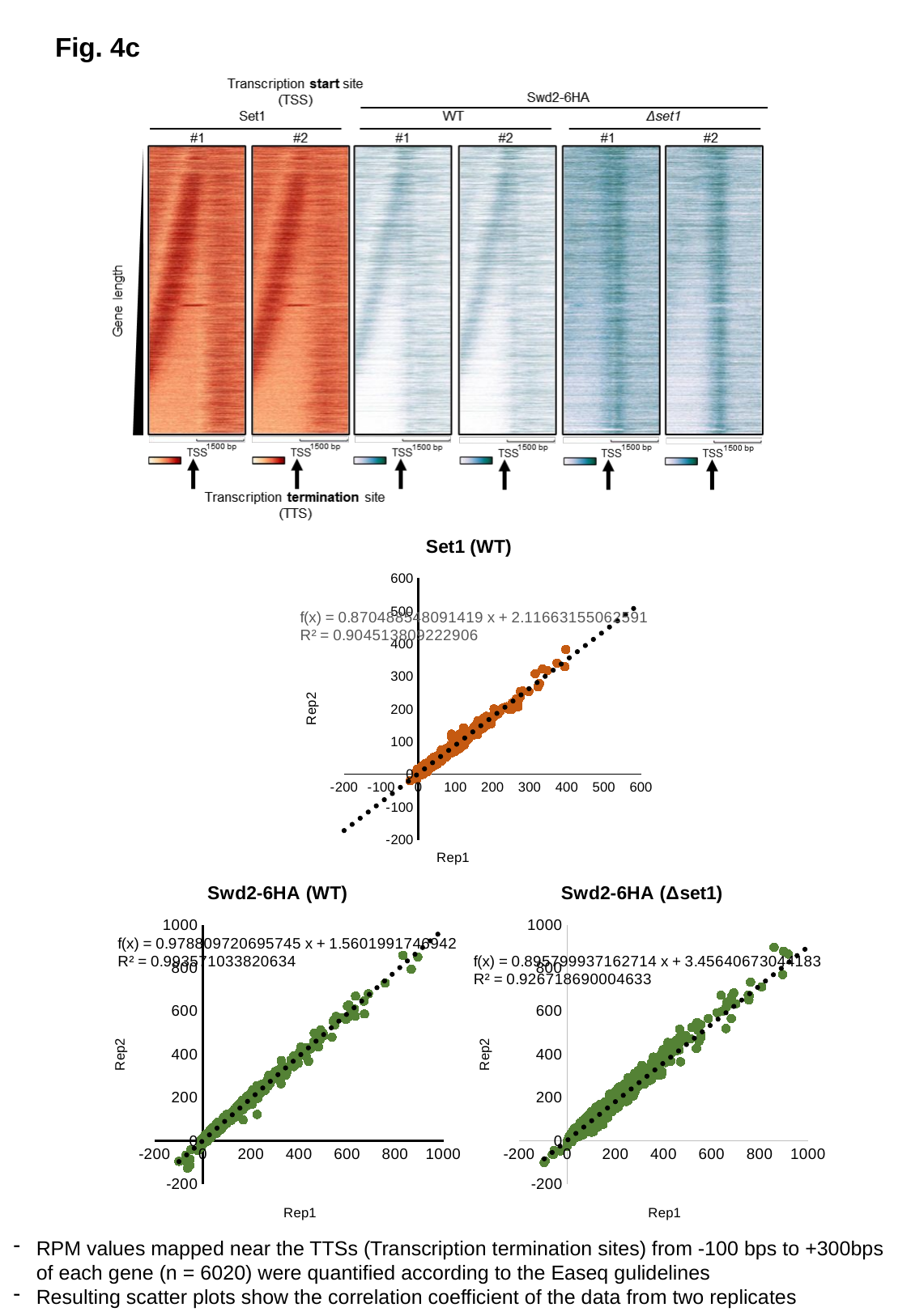

Fig. 4c
### Chart: Set1 (WT)
| Category | a-Set1_WT_minus_dset1_rep2 |
|---|---|
### Chart: Swd2-6HA (WT)
| Category | Swd2-6HA_minus_notag2 |
|---|---|
### Chart: Swd2-6HA (Δset1)
| Category | Swd2-6HA_dset1_minus_notag2 |
|---|---|RPM values mapped near the TTSs (Transcription termination sites) from -100 bps to +300bps of each gene (n = 6020) were quantified according to the Easeq gulidelines
Resulting scatter plots show the correlation coefficient of the data from two replicates

## Slide 9
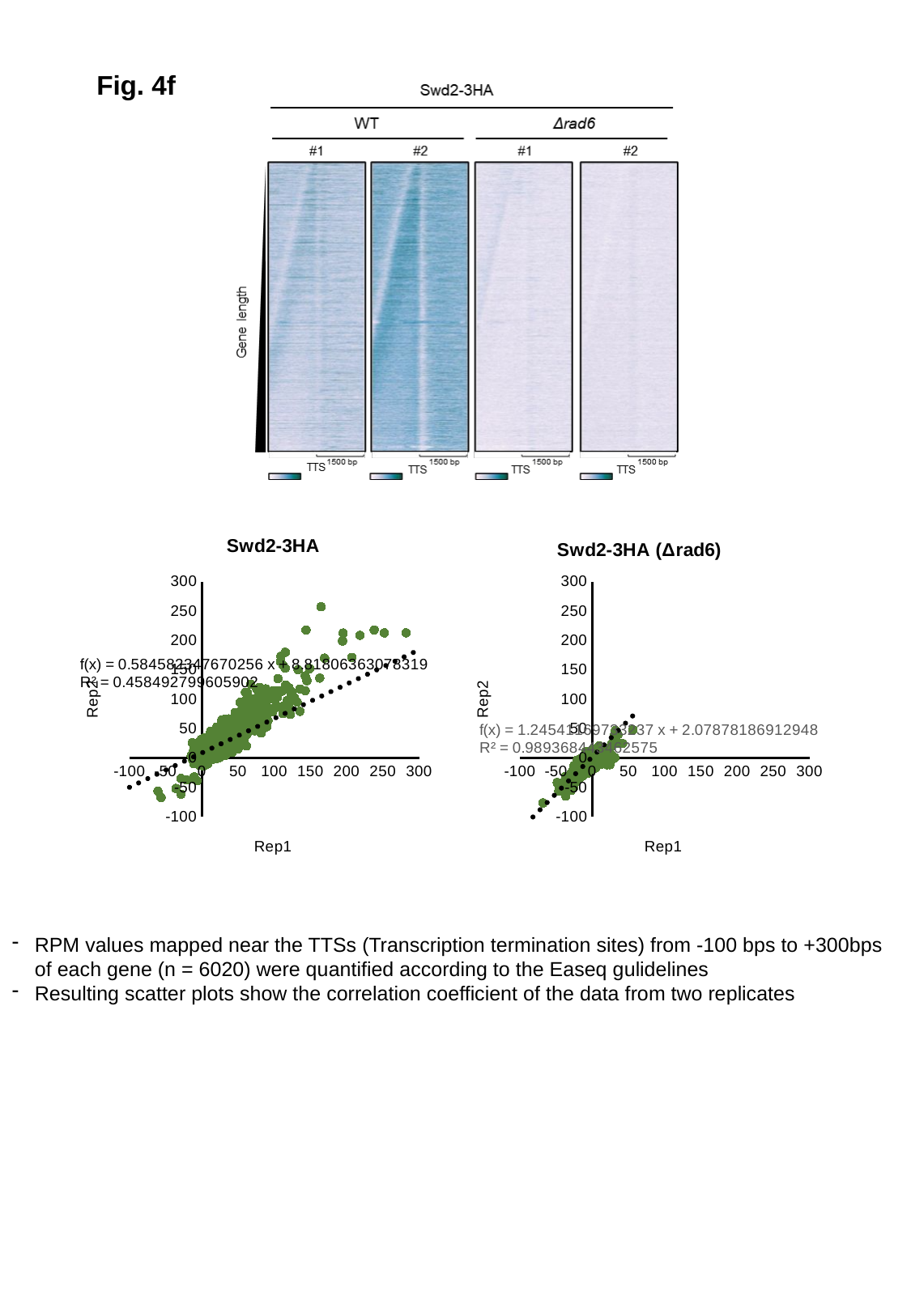

Fig. 4f
### Chart: Swd2-3HA (Δrad6)
| Category | Swd2-3HA_drad6_minus_notag_2 |
|---|---|
### Chart: Swd2-3HA
| Category | Swd2-3HA_minus_notag_2 |
|---|---|RPM values mapped near the TTSs (Transcription termination sites) from -100 bps to +300bps of each gene (n = 6020) were quantified according to the Easeq gulidelines
Resulting scatter plots show the correlation coefficient of the data from two replicates

## Slide 10
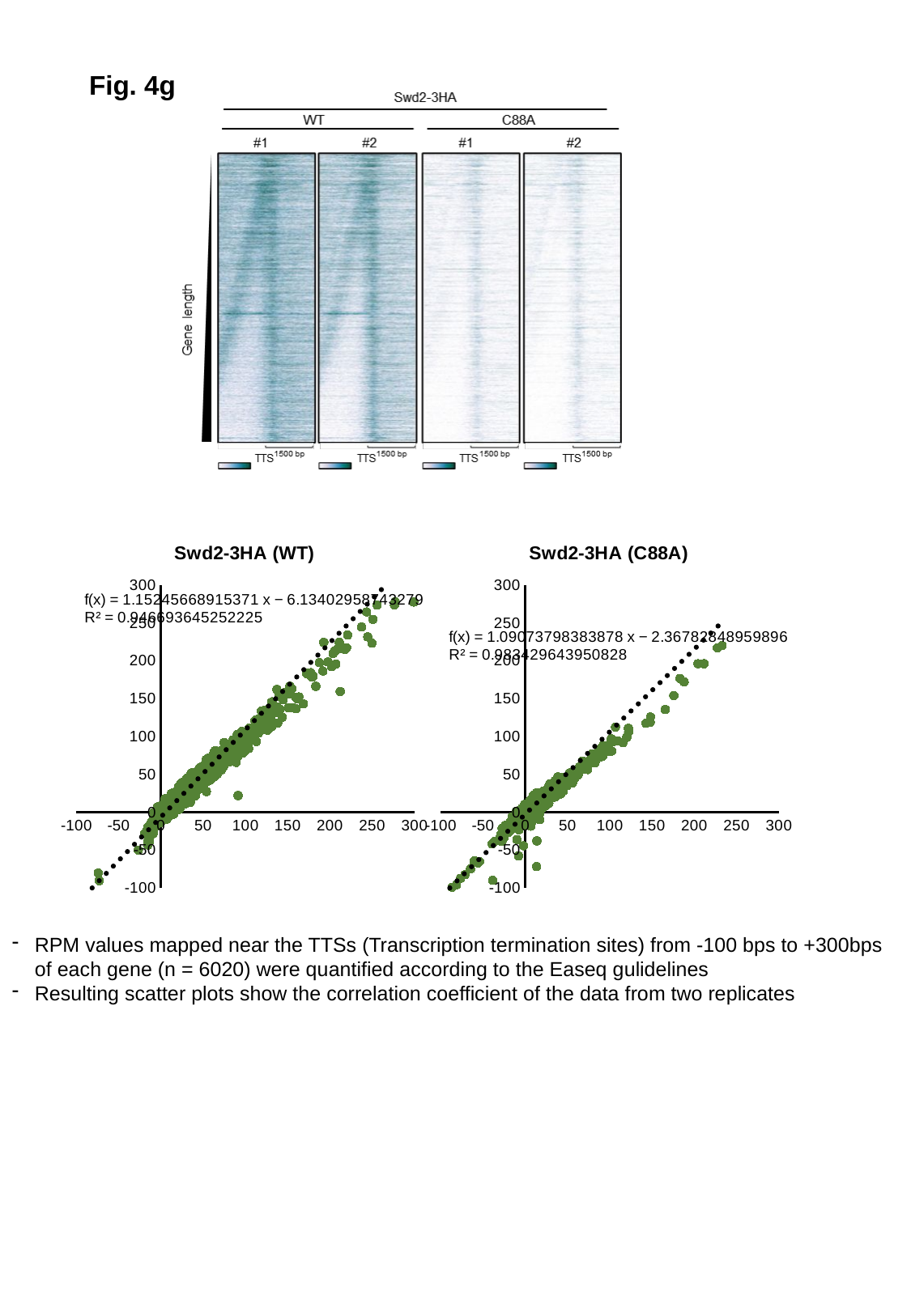

Fig. 4g
### Chart: Swd2-3HA (WT)
| Category | Swd2-3HA_Rad6_WT_minus_notag_2 |
|---|---|
### Chart: Swd2-3HA (C88A)
| Category | Swd2-3HA_C88A_minus_notag_2 |
|---|---|RPM values mapped near the TTSs (Transcription termination sites) from -100 bps to +300bps of each gene (n = 6020) were quantified according to the Easeq gulidelines
Resulting scatter plots show the correlation coefficient of the data from two replicates
