# Supplementary material for: Swd2/Cps35 determines H3K4 tri-methylation via interactions with Set1 and Rad6
Source: BMC Biol. 2024 May 3;22:105. doi: 10.1186/s12915-024-01903-3 (PMC11069235; doi:10.1186/s12915-024-01903-3)
Supplement: Supplementary file 3 — Additional file 3. Uncropped blots for western blot data. [file 12915_2024_1903_MOESM3_ESM.pptx]

## Slide 1
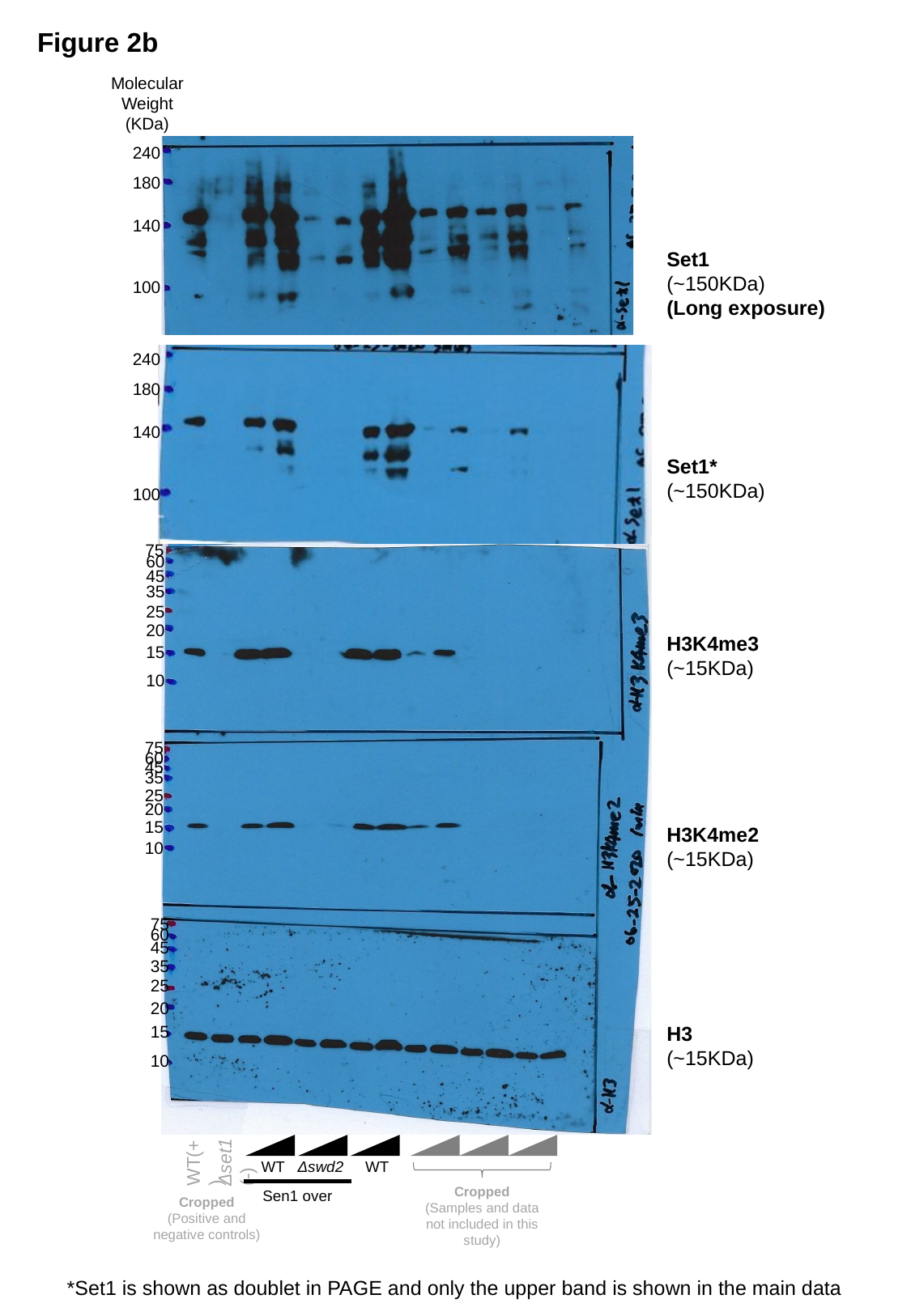

Figure 2b
Molecular Weight
(KDa)
240
180
140
Set1
(~150KDa)
(Long exposure)
100
240
180
140
Set1*
(~150KDa)
100
75
60
45
35
25
20
H3K4me3
(~15KDa)
15
10
75
60
45
35
25
20
15
H3K4me2
(~15KDa)
10
75
60
45
35
25
20
H3
(~15KDa)
15
10
Δset1 (-)
WT(+)
WT
Δswd2
WT
Cropped
(Samples and data not included in this study)
Sen1 over
Cropped
(Positive and negative controls)
*Set1 is shown as doublet in PAGE and only the upper band is shown in the main data

## Slide 2
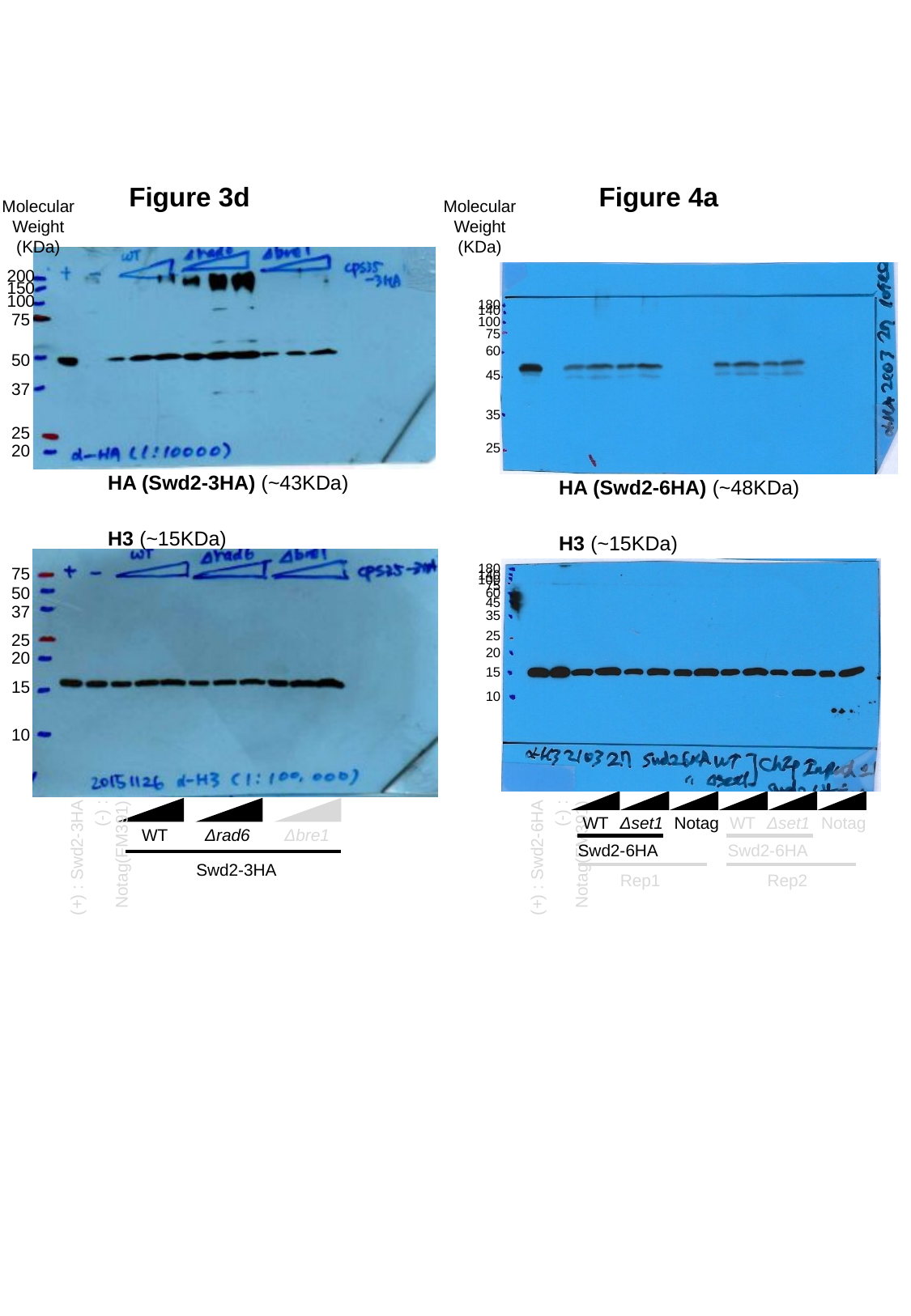

Figure 3d
Figure 4a
Molecular Weight
(KDa)
Molecular Weight
(KDa)
200
150
100
180
140
75
100
75
60
50
45
37
35
25
25
20
HA (Swd2-3HA) (~43KDa)
HA (Swd2-6HA) (~48KDa)
H3 (~15KDa)
H3 (~15KDa)
75
50
37
25
20
15
10
180
140
100
75
60
45
35
25
20
15
10
WT
Δset1
Notag
WT
Δset1
Notag
WT
Δrad6
Δbre1
Swd2-6HA
Swd2-6HA
(+) : Swd2-3HA
(+) : Swd2-6HA
(-) : Notag(FM391)
(-) : Notag(FM391)
Swd2-3HA
Rep1
Rep2

## Slide 3
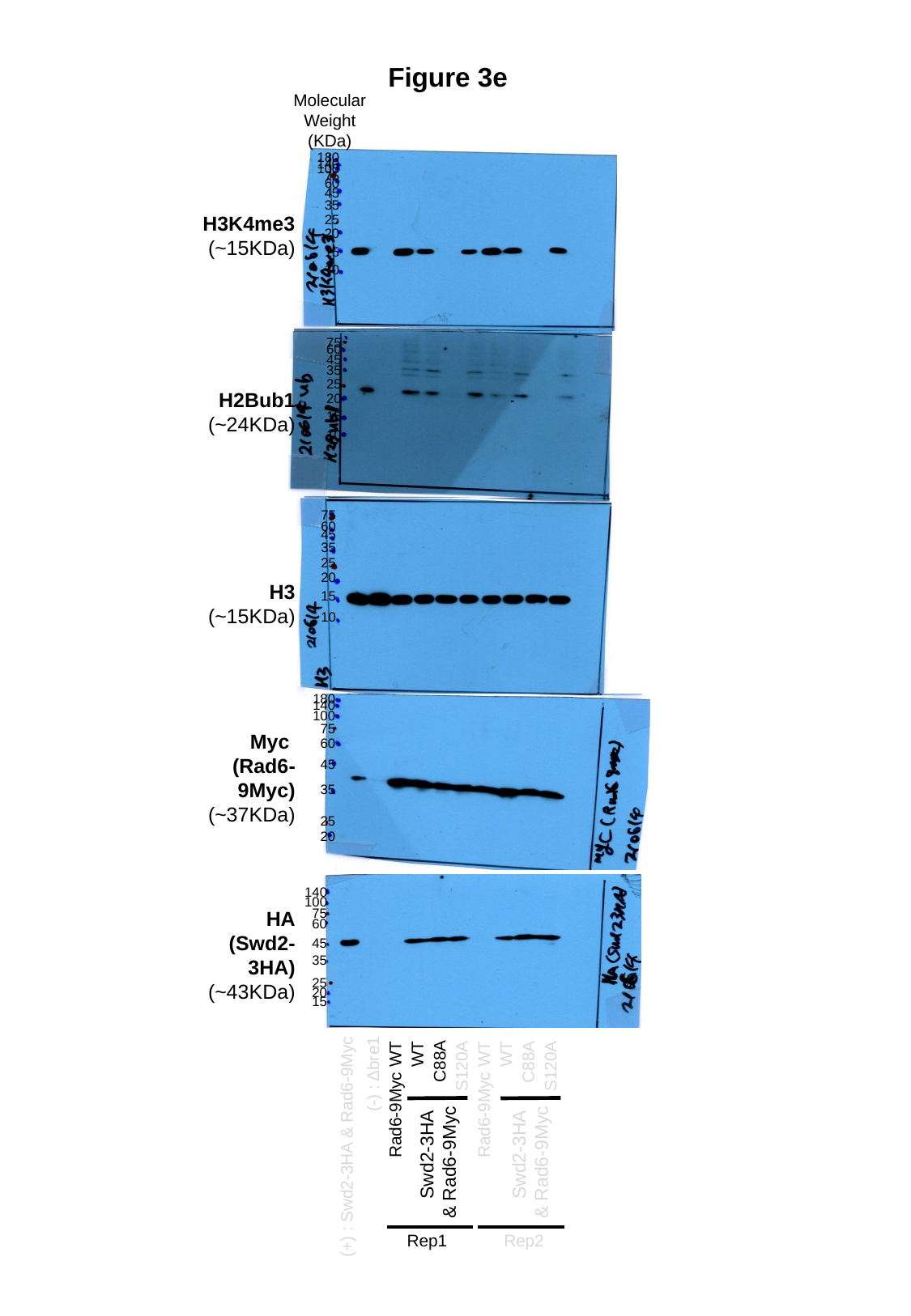

Figure 3e
Molecular Weight
(KDa)
180
140
100
75
60
45
35
H3K4me3
(~15KDa)
25
20
15
10
75
60
45
35
25
H2Bub1
(~24KDa)
20
15
10
75
60
45
35
25
20
H3
(~15KDa)
15
10
180
140
100
75
Myc
(Rad6-9Myc)
(~37KDa)
60
45
35
25
20
140
100
75
HA
(Swd2-3HA)
(~43KDa)
60
45
35
25
20
15
S120A
C88A
(-) : Δbre1
C88A
Rad6-9Myc WT
WT
Rad6-9Myc WT
WT
S120A
Swd2-3HA
& Rad6-9Myc
Swd2-3HA
& Rad6-9Myc
(+) : Swd2-3HA & Rad6-9Myc
Rep1
Rep2
