# Supplementary material for: Swd2/Cps35 determines H3K4 tri-methylation via interactions with Set1 and Rad6
Source: BMC Biol. 2024 May 3;22:105. doi: 10.1186/s12915-024-01903-3 (PMC11069235; doi:10.1186/s12915-024-01903-3)
Supplement: Supplementary file 4 — Additional file 4. Reads of spikes-in chromatin mapped to the genome of C. albicans (for Set1 ChIP-seq) or S. pombe (other ChIP-seqs). [file 12915_2024_1903_MOESM4_ESM.pptx]

## Slide 1
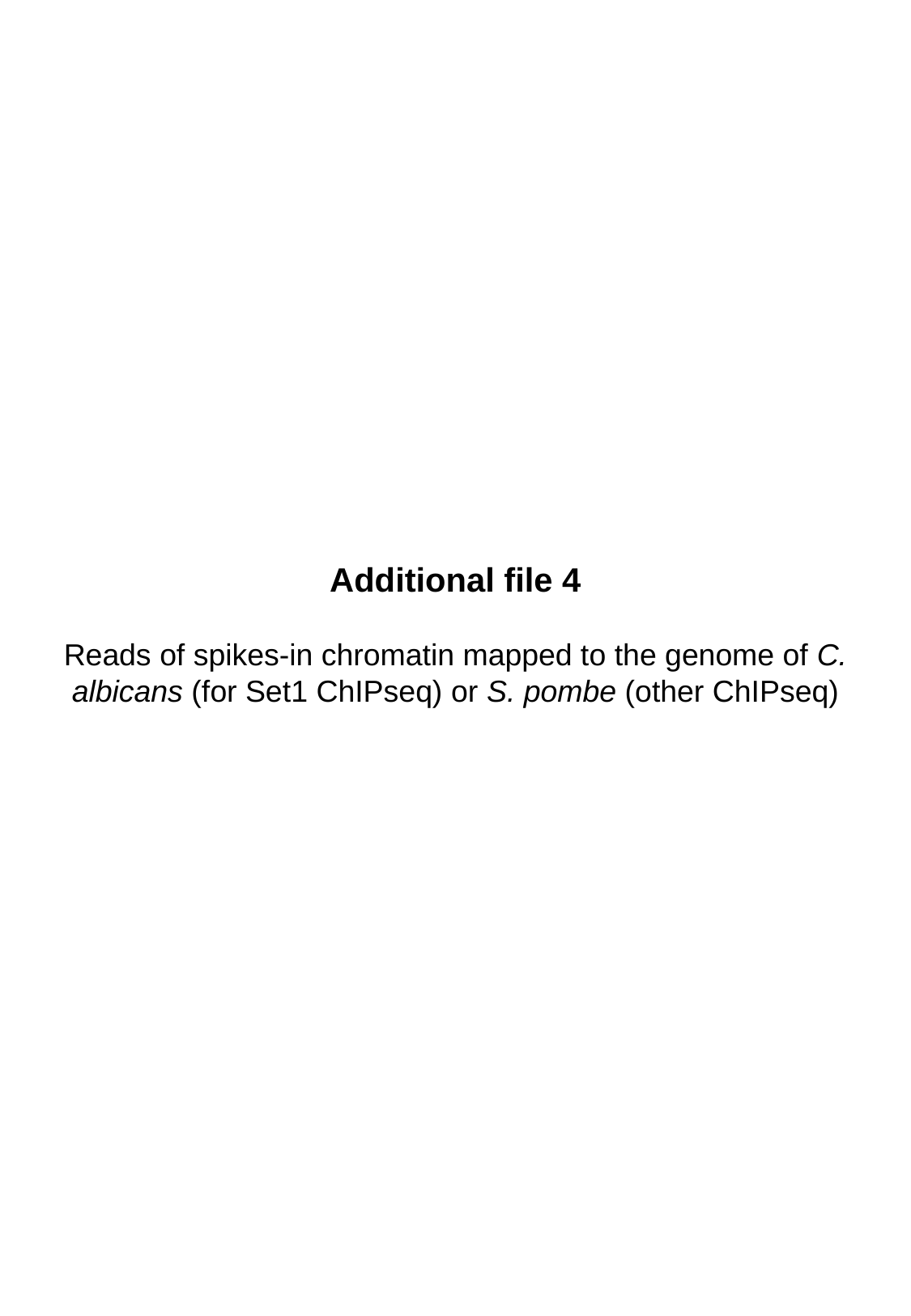

Additional file 4
Reads of spikes-in chromatin mapped to the genome of C. albicans (for Set1 ChIPseq) or S. pombe (other ChIPseq)

## Slide 2
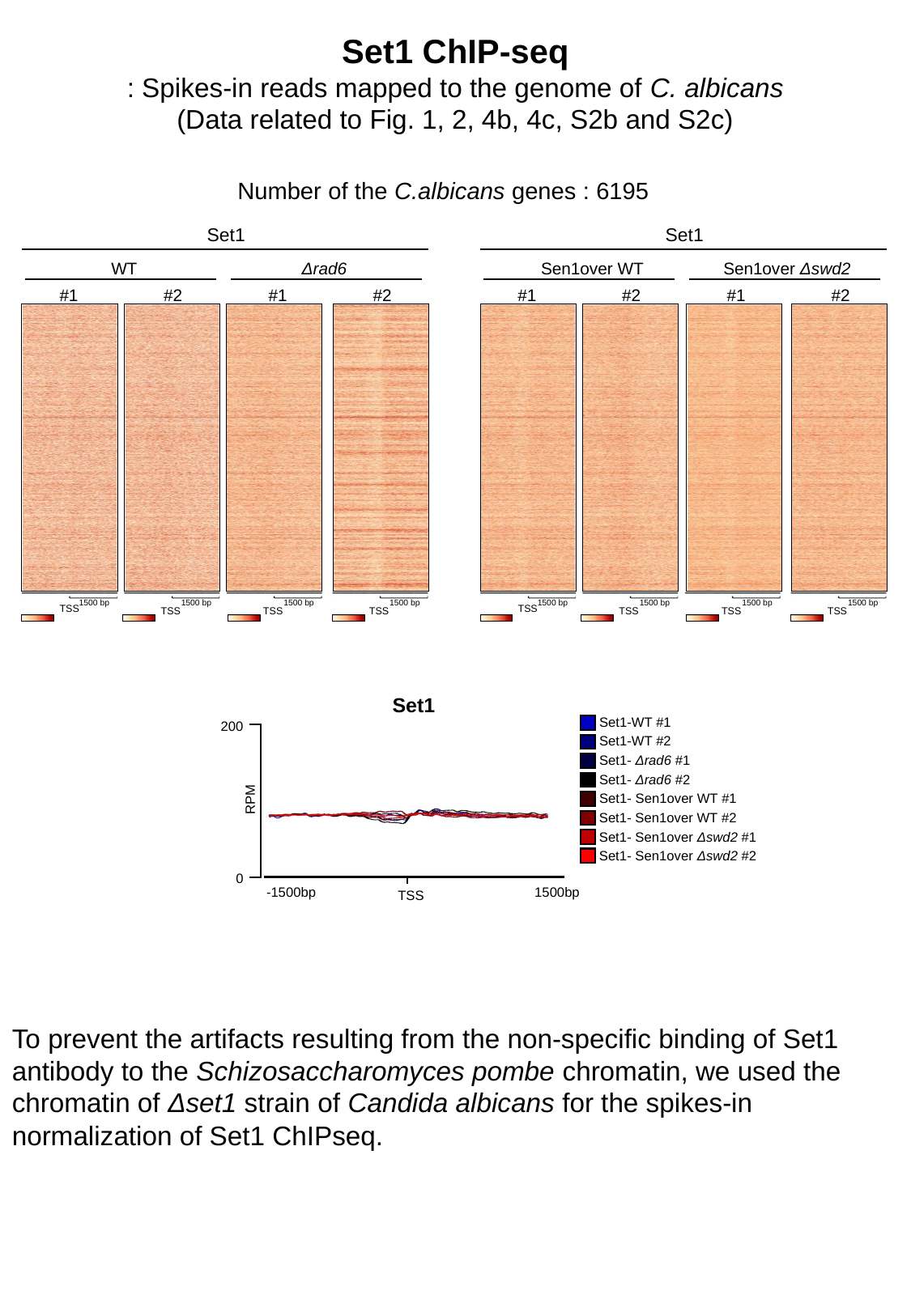

Set1 ChIP-seq
: Spikes-in reads mapped to the genome of C. albicans
(Data related to Fig. 1, 2, 4b, 4c, S2b and S2c)
Number of the C.albicans genes : 6195
Set1
Set1
WT
Δrad6
Sen1over WT
Sen1over Δswd2
#1
#2
#1
#2
#1
#2
#1
#2
1500 bp
TSS
1500 bp
TSS
1500 bp
TSS
1500 bp
TSS
1500 bp
TSS
1500 bp
TSS
1500 bp
TSS
1500 bp
TSS
Set1
Set1-WT #1
200
Set1-WT #2
Set1- Δrad6 #1
Set1- Δrad6 #2
Set1- Sen1over WT #1
RPM
Set1- Sen1over WT #2
Set1- Sen1over Δswd2 #1
Set1- Sen1over Δswd2 #2
0
-1500bp
1500bp
TSS
To prevent the artifacts resulting from the non-specific binding of Set1 antibody to the Schizosaccharomyces pombe chromatin, we used the chromatin of Δset1 strain of Candida albicans for the spikes-in normalization of Set1 ChIPseq.

## Slide 3
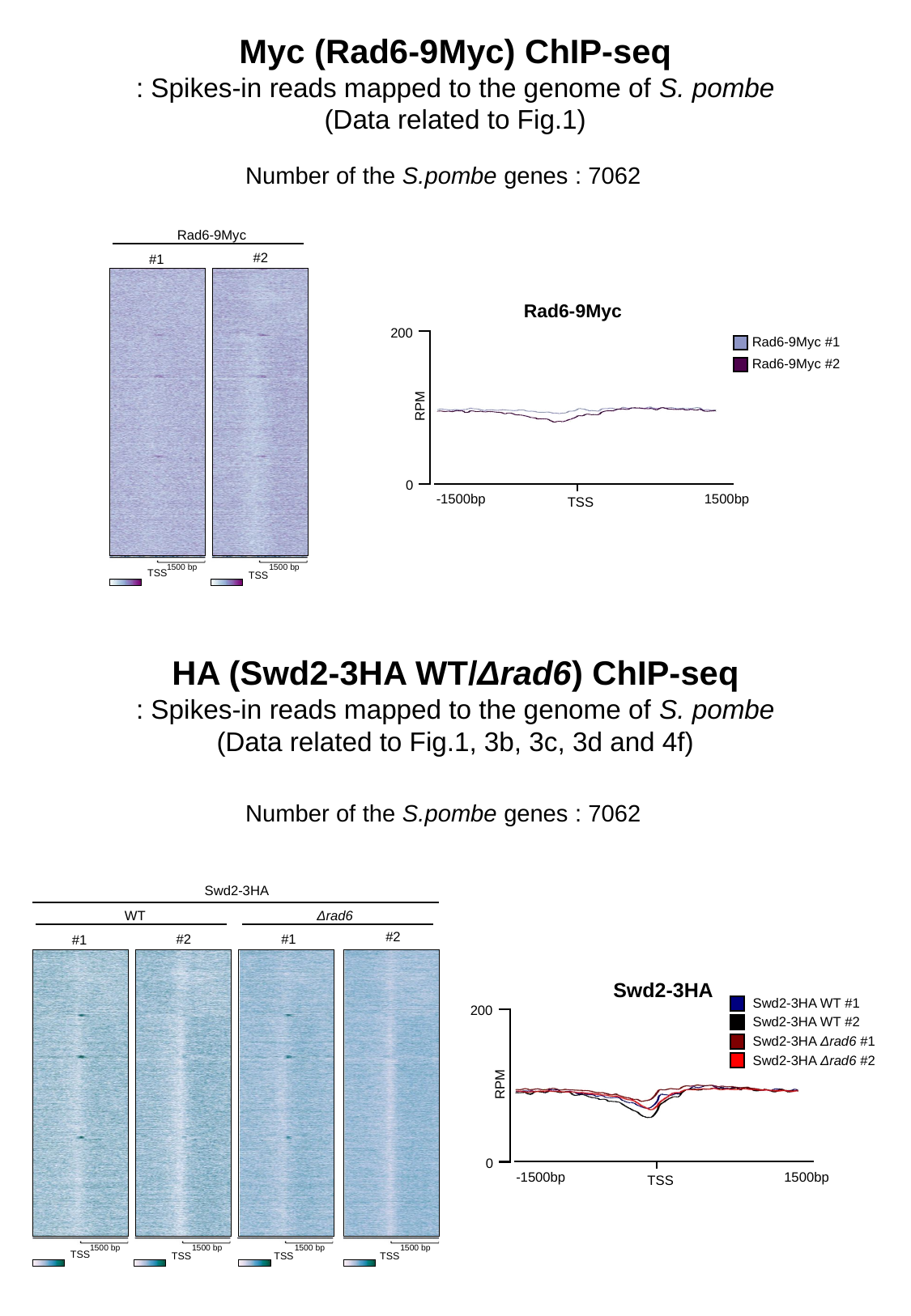

Myc (Rad6-9Myc) ChIP-seq
: Spikes-in reads mapped to the genome of S. pombe
(Data related to Fig.1)
Number of the S.pombe genes : 7062
Rad6-9Myc
#2
#1
Rad6-9Myc
200
Rad6-9Myc #1
Rad6-9Myc #2
RPM
0
-1500bp
1500bp
TSS
1500 bp
TSS
1500 bp
TSS
HA (Swd2-3HA WT/Δrad6) ChIP-seq
: Spikes-in reads mapped to the genome of S. pombe
(Data related to Fig.1, 3b, 3c, 3d and 4f)
Number of the S.pombe genes : 7062
Swd2-3HA
WT
Δrad6
#2
#2
#1
#1
Swd2-3HA
Swd2-3HA WT #1
Swd2-3HA WT #2
Swd2-3HA Δrad6 #1
Swd2-3HA Δrad6 #2
200
RPM
0
-1500bp
1500bp
TSS
1500 bp
TSS
1500 bp
TSS
1500 bp
TSS
1500 bp
TSS

## Slide 4
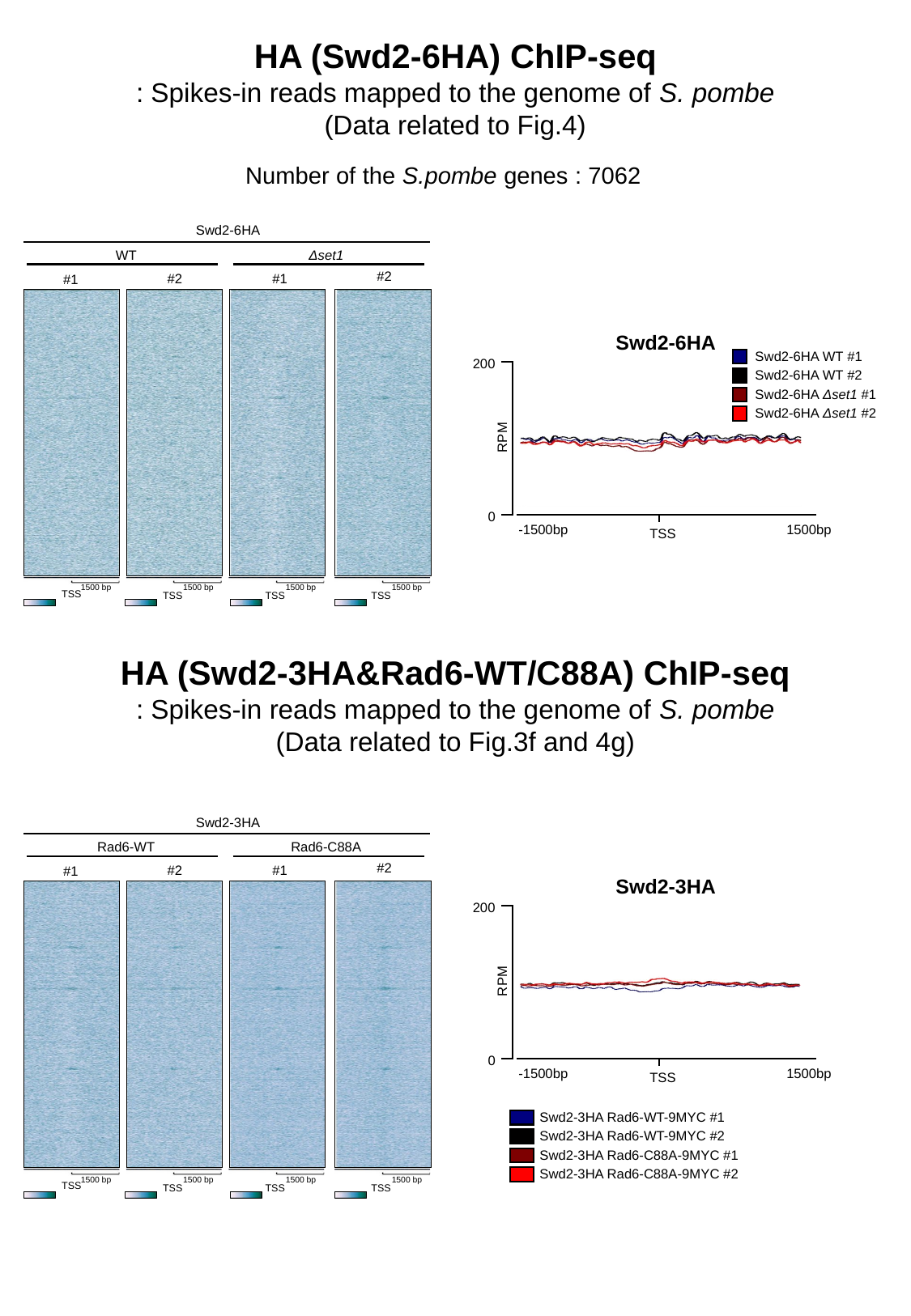

HA (Swd2-6HA) ChIP-seq
: Spikes-in reads mapped to the genome of S. pombe
(Data related to Fig.4)
Number of the S.pombe genes : 7062
Swd2-6HA
WT
Δset1
#2
#2
#1
#1
Swd2-6HA
Swd2-6HA WT #1
Swd2-6HA WT #2
Swd2-6HA Δset1 #1
Swd2-6HA Δset1 #2
200
RPM
0
-1500bp
1500bp
TSS
1500 bp
TSS
1500 bp
TSS
1500 bp
TSS
1500 bp
TSS
HA (Swd2-3HA&Rad6-WT/C88A) ChIP-seq
: Spikes-in reads mapped to the genome of S. pombe
(Data related to Fig.3f and 4g)
Swd2-3HA
Rad6-WT
Rad6-C88A
#2
#2
#1
#1
Swd2-3HA
200
RPM
0
-1500bp
1500bp
TSS
Swd2-3HA Rad6-WT-9MYC #1
Swd2-3HA Rad6-WT-9MYC #2
Swd2-3HA Rad6-C88A-9MYC #1
Swd2-3HA Rad6-C88A-9MYC #2
1500 bp
TSS
1500 bp
TSS
1500 bp
TSS
1500 bp
TSS
