# Supplementary material for: Swd2/Cps35 determines H3K4 tri-methylation via interactions with Set1 and Rad6
Source: BMC Biol. 2024 May 3;22:105. doi: 10.1186/s12915-024-01903-3 (PMC11069235; doi:10.1186/s12915-024-01903-3)
Supplement: Supplementary file 5 — Additional file 5: Table S1. Strains used in this study. [file 12915_2024_1903_MOESM5_ESM.docx]

**Table 1**. Strains used in this study

| Strains | Genotype | Reference |
| --- | --- | --- |
| FM391 (BY4741) | MATa his3Δ0 leu2 Δ0 met15Δ0 ura3Δ0 | [1] |
| Δrad6 (JL294) | FM391-Δrad6::KanMX | [2] |
| FY23 (JL220) | MATa ura3-52 leu2Δ1 trp1Δ63 | [3] |
| Rad6-9Myc (JL685) | JL220-Rad6::9Myc::TRP | This study |
| FY23 + pRS316-Gal1p-FLAG-Swd2 (JL1172) | JL220 (pRS316-Gal1p-FLAG-Swd2) | This study |
| Rad6-9Myc + pRS316-Gal1p-FLAG-Swd2 (JL1171) | JL220-Rad6::9Myc::TRP (pRS316-Gal1p-FLAG-Swd2) | This study |
| Rad6-C88A-9Myc + pRS316-Gal1p-FLAG-Swd2 (JL1173) | JL220-Rad6-C88A::9Myc::TRP (pRS316-Gal1p-FLAG-Swd2) | This study |
| 551 wild type (JL150) | MATa ura3-52 his3∆200 ade2-101(ochre) lys2-80 (1m) trp-1∆1 leu2-∆1 TEladh4:L:URA3(VIIL Telomere) | [2] |
| Swd2-3HA in WT (JL003) | JL150-Swd2::3HA::HIS | [2] |
| Swd2-6HA in WT (JL1205) | JL150-Swd2::6HA::TRP | This study |
| Swd2-6HA in Δset1 (JL1207) | JL150-Swd2::6HA::TRP- Δset1::HIS | This study |
| Swd2-3HA in Δrad6 (JL004) | JL150-Swd2::3HA::HIS-Δrad6::KanMX | [2] |
| Swd2-3HA &Rad6-9myc (JL1188) | JL150-Swd2::3HA::HIS-Rad6::9Myc::TRP | This study |
| Swd2-3HA &Rad6-C88A-9myc (JL1189) | JL150-Swd2::3HA::HIS-Rad6-C88A::9Myc::TRP | This study |
| Sen1 overexpressed WT (JL144) | WT strain with overexpressing Sen1 C-terminal fragment | [4] |
| Sen1 overexpressed ∆swd2 (JL145) | Swd2 deletion mutant with overexpressing Sen1 C-terminal fragment | [4] |
| *Schizosaccharomyces pombe* strain 972 h- (ATCC 24843) | Wildtype strain | [5] |
| *Candida albicans*  Set1 null mutant (∆SETdU) | ∆set1::hisG/∆set1::hisG ∆ura3::imm434/URA3 | [6] |

1. Brachmann CB, Davies A, Cost GJ, Caputo E, Li J, Hieter P, Boeke JD: **Designer deletion strains derived from Saccharomyces cerevisiae S288C: a useful set of strains and plasmids for PCR-mediated gene disruption and other applications**. *Yeast* 1998, **14**(2):115-132.

2. Lee JS, Shukla A, Schneider J, Swanson SK, Washburn MP, Florens L, Bhaumik SR, Shilatifard A: **Histone crosstalk between H2B monoubiquitination and H3 methylation mediated by COMPASS**. *Cell* 2007, **131**(6):1084-1096.

3. Winston F, Dollard C, Ricupero-Hovasse SL: **Construction of a set of convenient Saccharomyces cerevisiae strains that are isogenic to S288C**. *Yeast* 1995, **11**(1):53-55.

4. Nedea E, Nalbant D, Xia D, Theoharis NT, Suter B, Richardson CJ, Tatchell K, Kislinger T, Greenblatt JF, Nagy PL: **The Glc7 phosphatase subunit of the cleavage and polyadenylation factor is essential for transcription termination on snoRNA genes**. *Mol Cell* 2008, **29**(5):577-587.

5. Leupold U: **Die Vererbung von Homothallie und Heterothallie bei Schizosaccharomyces Pombe**. Copenhague; 1950.

6. Raman SB, Nguyen MH, Zhang Z, Cheng S, Jia HY, Weisner N, Iczkowski K, Clancy CJ: **Candida albicans SET1 encodes a histone 3 lysine 4 methyltransferase that contributes to the pathogenesis of invasive candidiasis**. *Mol Microbiol* 2006, **60**(3):697-709.
